# Characterization of a GH10 extremely thermophilic xylanase from the metagenome of hot spring for prebiotic production

**DOI:** 10.1038/s41598-023-42920-6

**Published:** 2023-09-25

**Authors:** Yi-Rui Yin, Xin-Wei Li, Chao-Hua Long, Lei Li, Yu-Ying Hang, Meng-Di Rao, Xin Yan, Quan-Lin Liu, Peng Sang, Wen-Jun Li, Li-Quan Yang

**Affiliations:** 1 College of Agriculture and Biological Science, Dali University, Dali, 671003 People’s Republic of China; 2 Key Laboratory of Bioinformatics and Computational Biology, Department of Education of Yunnan Province, Dali University, Dali, 671003 People’s Republic of China; 3 State Key Laboratory of Biocontrol, Guangdong Provincial Key Laboratory of Plant Resources and Southern Marine Science and Engineering Guangdong Laboratory (Zhuhai), School of Life Sciences, Sun Yat-Sen University, Guangzhou, 510275 People’s Republic of China

**Keywords:** Biochemistry, Biological techniques, Biotechnology

## Abstract

A xylanase gene (named *xyngmqa*) was identified from the metagenomic data of the Gumingquan hot spring (92.5 °C, pH 9.2) in Tengchong City, Yunnan Province, southwest China. It showed the highest amino acid sequence identity (82.70%) to endo-1,4-beta-xylanase from *Thermotoga caldifontis*. A constitutive expression plasmid (denominated pSHY211) and double-layer plate (DLP) method were constructed for cloning, expression, and identification of the XynGMQA gene. The XynGMQA gene was synthesized and successfully expressed in *Escherichia coli* DH5α. XynGMQA exhibited optimal activity at 90 °C and pH 4.6, being thermostable by maintaining 100% of its activity after 2 h incubated at 80 °C. Interestingly, its enzyme activity was enhanced by high temperatures (70 and 80 °C) and low pH (3.0–6.0). About 150% enzyme activity was detected after incubation at 70 °C for 20 to 60 min or 80 °C for 10 to 40 min, and more than 140% enzyme activity after incubation at pH 3.0 to 6.0 for 12 h. Hydrolytic products of beechwood xylan with XynGMQA were xylooligosaccharides, including xylobiose (X2), xylotriose (X3), and xylotetraose (X4). These properties suggest that XynGMQA as an extremely thermophilic xylanase, may be exploited for biofuel and prebiotic production from lignocellulosic biomass.

Hemicellulose, as the second largest component of lignocellulosic biomass, with a ratio of 28.5–37.2%^1^, is widely used in the production of biofuels and bio-based chemicals^2^. Xylan, one of the main constituents of hemicellulose, is composed of xylose monomers bound by β-1,4-glycoside bonds. Complete degradation of xylan requires the involvement of endo-1,4-beta-xylanase and beta-xylosidase. In recent years, xylanases have been generally used in various industries, such as biofuels, human food, animal feed, pulp, prebiotic production, etc.^3,4^.

In nature, xylanases are broadly distributed in fungi, bacteria, and archaea, such as *Trichoderma reesei*^5^, *Thermoactinospora rubra* YIM 77501^T^^6^, and *Thermococcus zilligii*^7^. However, many xylanases from common environmental microorganisms are limited by their thermal stability when used in specific industries. Thermophilic xylanases are mainly derived from thermophilic microorganisms, which are primarily distributed in thermal environments, such as hot springs, hydrothermal vents, and compost^8,9^. Due to the limitations of current laboratory pure culture techniques, more than 99% of prokaryotic microorganisms cannot be cultured, which limits the development of thermophilic xylanases^10^. Metagenomic technology, which can directly obtain nucleotide sequences of most genes from environmental DNA, is not limited by cultural technology^11^. Therefore, metagenomic technology has great potential to exploit thermophilic xylanase from uncultured extreme environmental microorganisms^12^. 

Here, a novel xylanase gene (*xyngmqA*) was identified from metagenomic data of the Gumingquan hot spring in Rehai area of Tengchong City, southwest China. This gene sequence was synthesized artificially, and the constitutive expression plasmid (denominated pSHY211) was constructed for cloning and expression of the XynGMQA gene in *Escherichia coli* DH5α. The activity of recombinant enzymes was characterized after heterologous expression and protein purification. The results show that XynGMQA, an extremely thermophilic xylanase, may be exploited for biofuel production from lignocellulosic biomass.

## Materials and methods

### Strains and medium

*Escherichia coli* DH5α was used for xylanase gene clone and expression. *E. coli* strains were grown on LB medium with 100 μg/mL Kanamycin. DNA isolation and purification kits were purchased from Sangon, China.

### Construction of constitutive expression plasmid pSHY211

**Figure 1:**
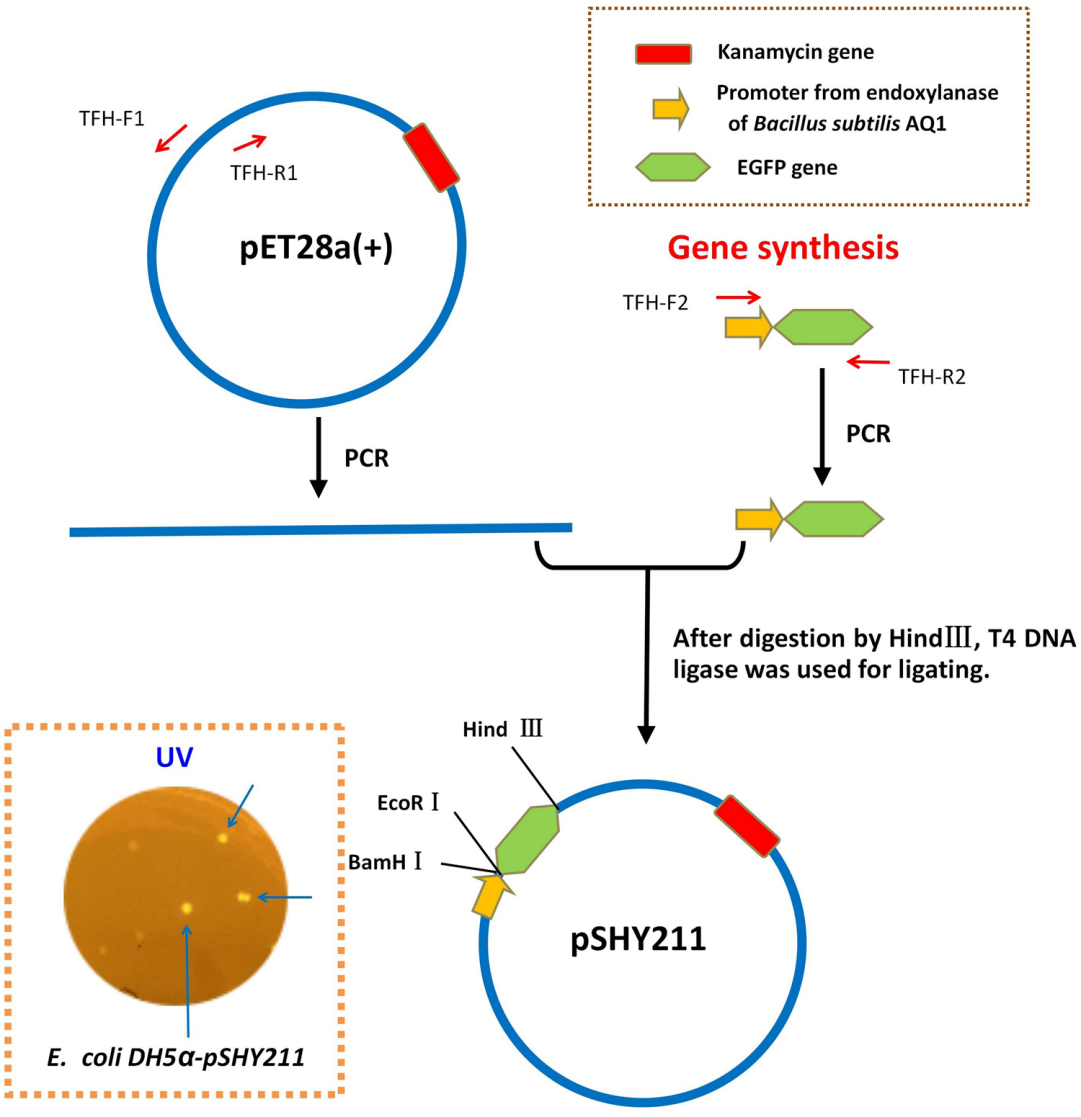
Construction process of constitutive expression plasmid pSHY211.

The construction process of recombinant plasmid was shown in Fig. 1. In this study, all PCR processes were performed by TransStarFastPfu Fly DNA Polymerase (TransGen Biotech, China). The PCR program consisted of denaturation at 95 °C for 3 min, followed by 32 cycles at 98 °C for 20 s, 65 °C for 30 s, and 72 °C for 1 min (the target DNA fragment ≤ 2000 bp) or 4 min (4000 bp ≤ the target DNA fragment ≤ 8000 bp), and then a final incubation at 72 °C for 5 min for the final extension. Using *pET*28a as a template, TFH-F1 and THF-R1 primers (Supplementary Table S1) were used to obtain TFH1 fragment (5194 bp). DNA fragment (named TFH2) containing promoter of GH11 endo-xylanase gene (GenBank NO.: FJ644630.1) from *Bacillus subtilis* AQ1^13^ and EGFP gene sequences were obtained by gene synthesis. PCR was performed by TFH-F2 and THF-R2 primers (Supplementary Table S1). Then TFH1 and TFH2, which had been previously digested by *Hind* III (Thermo Scientific, USA), were linked with T4 DNA ligase (TransGen Biotech, China). DNA linkage products were transformed into *E. coli* DH5α to obtain constitutive expression plasmid pSHY211.

### Sample collection and metagenomic sequencing

Sandy soil samples were collected from Gumingquan hot spring (GMQ) in the Rehai area of Tengchong City, Yunnan Province, China. Coordinates are latitude 24.95002°N, longitude 98.43729°E. The surface temperature of Gumingquan hot spring was around 92.5 °C with a pH of 9.2. DNA isolation of the soil sample was performed with the Power Soil Kit (MOBIO DNeasy PowerSoil Kit, USA) according to the operating manual. Metagenomic sequencing was performed by HiSeq 2500 instrument at GENWIZ, Suzhou. The Velvet assembly program version 1.2.08 used assembly sequences^14^. The IMG server (https://img.jgi.doe.gov/cgi-bin/mer/main.cgi) was used to investigate the sequences. To further analyze the potential functions of individual genes and ORFs, KEGG^15^, COG^16^, and Pfam^17^ were employed.

### Synthesis and sequence analysis of the XynGMQA gene

The function prediction analysis was performed with KEGG and COG, and functional genes of xylanases were screened, and their domains were analyzed by Pfam database. In this study, only one new xylanase gene sequence (named *xyngmqa*) with complete domain was obtained from the GMQ metagomic database, which belongs to the GH10 family. The nucleotide sequence of the xylanase gene was submitted to GenBank under accession number MW131969. The XynGMQA gene was synthesized according to *E. coli* base preference (Supplementary Figure S1) and cloned to the pUC18 vector. BLASTx and BLASTp programs (http://blast.ncbi.nlm.nih.gov/Blast.cgi) were used to align XynGMQA DNA and protein sequences, respectively. Signal peptides were predicted using SignalP (https://services.healthtech.dtu.dk/service.php?SignalP-5.0). Primary structures of amino acid sequences were deduced and analyzed using EXPASY tools (http://web.expasy.org/protparam). The protein sequence of XynGMQA was compared in NCBI BLASTp, and the xylanase sequences of the same genus or neighboring genera (*Thermotoga* and *Pseudothermotoga*) with high similarity were selected as neighboring sequences, and the xylanase protein sequences of other genera with low similarity in the comparison were selected as outgroups for constructing the multiple alignment and phylogenetic tree. Multiple alignments with closely related XynGMQA protein sequences were performed using Clustal X^18^. Phylogenetic analyses were performed using the MEGA 7 software package^19^. The phylogenetic tree was constructed using the maximum likelihood (ML) method and Poisson modified model. XynGMQA sequence was compared with protein structure data from the protein data bank (http://www.rcsb.org). A structural model of XynGMQA was generated with the MODELLER package^20^ using endo-β-1, 4-xylanase (PDB ID, 1VBU; sequence identity, 75.62%) from *Thermotoga maritima* as the template. Multiple sequence alignment was performed by Clustal X version 2.0^21^ and Espript 3 (http://espript.ibcp.fr/ESPript/cgi-bin/ESPript.cgi).

### Cloning and identification of XynGMQA

XynGMQA gene was amplified by PCR using the following primers: XynGMQA-F (CATCATCATCATCATCATGAACTGAAAAGTCTGCGTCTGGTTAT) and XynGMQA-R (GTGCTCGAGTGCGGCCGCAAGTCATTTCTGGCGCTCCATGACTT) from recombinant plasmid PUC18-XynGMQA. The underlined sequence represents a homologous recombinant fragment of the pSHY211 vector that was previously digested with *EcoR* I and *Hind* III. The PCR program consisted of denaturation at 95 °C for 3 min, followed by 30 cycles at 98 °C for 15 s, 60 °C for 20 s, and 72 °C for 30 s, and then a final incubation at 72 °C for 5 min for the final extension. The PCR product was inserted into pSHY211 using the *pEASY-Uni* Seamless Cloning and Assembly Kit (TransGen Biotech, China) to yield the expression plasmid pSHY211-XynGMQA.

The xylanase activity detection of *E. coli* clones containing recombinant plasmid was identified by double-layer plate (DLP) method. The first layer of culture medium (LB medium with 100 μg/mL kanamycin, 2% agar) was used to culture *E. coli* clones at 37 °C for 16 h. Then, the second medium layer (PBS buffer containing 1% agarose, 0.1% xylan, 0.05% congo red, 1 mg/mL lysozyme, pH 8.0) was added to completely cover the *E. coli* clones. After incubation at 37 °C for 6 h, the colony transparency of the *E. coli* clone was observed. Finally, the recombinant *E. coli* clones with transparent zones were identified, sequenced, and heterologously expressed.

### Expression and purification of XynGMQA

The recombinant *E. coli* DH5α with pSHY211-XynGMQA was cultured in 200 mL LB broth containing 100 µg/mL kanamycin at 37 °C with shaking at 180 rpm for 8 h. The culture was then incubated at 25 °C with shaking at 180 rpm for 12 h. *E. coli* biomass was collected by centrifugation at 12,000 × g, 4 °C for 15 min, and cell lysates were ultrasonically collected. After centrifugation, cell-free extracts were purified using the Ni-chelating affinity column (Histrap, TransGen Biotech, China) according to the method previously reported by Yin et al.^6^. The purified proteins were desalted using disposable D-10 Desalting Columns (GE, USA). The desalted protein was detected using 10% SDS-PAGE. Protein concentrations were determined with Bradford Protein Assay Kit (Order NO. C503031, Sangon Biotech, China) using bovine serum albumin as the standard.

### Xylanase assay

The recombinant XynGMQA activity was determined using the method reported by Yin et al.^6^. DNS (3,5-dinitrosalicylic acid) method was used to determine reducing sugars^22^. One unit (U) of xylanase activity was identified as the amount of enzyme releasing 1 µmol reducing sugar per min.

### Biochemical characterization

The optimum pH for XynGMQA was investigated in buffer pHs ranging from 3.0 to 9.0 (Na_2_HPO_4_-citrate buffer, pH 3.0–8.0; borate buffer, pH 7.6–9.0). The optimum temperature for XynGMQA was measured from 30 °C to 100 °C at optimum pH. To assess thermostability and pH stability, the residual xylanase activities were determined after incubating the XynGMQA at different temperatures (70, 80, and 90 °C) for different times (0, 20, 40, 60, 80, 100, and 120 min) and pH 3.0 to 9.0 for 12 h, respectively. To evaluate the effects of metal ions and chemical reagents on XynGMQA activity, 10 mM of various metal ions, 1% of detergents or enzyme inhibitors, 10% of ionic liquid (1-allyl-3-methylimidazolium chloride), and alcohol were added individually to the reaction system. The control was tested using the same process described above without any additives to the reaction mixture.

To investigate the substrate specificity of XynGMQA, beechwood xylan, oat xylan, avicel (or microcrystalline cellulose), beta-(1,3;1,4)-glucan, CMC (Sodium carboxymethylcellulose), soluble starch and, *p*NPX (*p*-nitrophenyl β-D-xylopyranoside), were used as substrates (1%, w/v) to measure enzymatic activity. The kinetic constants of XynGMQA were determined using different concentrations of beechwood xylan (0.1 to 20 mg/mL) at optimum pH and temperature for 5 min. The *Km* (Michaelis–Menten constant) and *Vmax* (maximum velocity) were calculated by the Lineweaver–Burk plot.

### TLC analysis

To analyze the hydrolytic product of xylan by XynGMQA, a reaction mixture consisting of 1% beech xylan and 10 μg of purified XynGMQA was incubated at 90° C for 1 h. Hydrolytic products of beechwood xylan were characterized by TLC (thin-layer chromatography) with Silica gel60 Glass plates (Merck, Darmstadt, Germany). The spreading solution was 1-butanol/acetic acid/water (2:1:1, v/v/v). The sugar was detected by treating at 120 °C for 10 min after spraying the TLC plate with freshly prepared 5% (v/v) H_2_SO_4_ in ethanol. The sugar standards used in this study were X1 (xylose), X2 (xylobiose), X3 (xylotriose), and X4 (xylotetraose).

### Statistical analysis

Unless otherwise stated, all assays were triplicated and the mean was used in all analyses. The results were analyzed by SPSS 20.0 and expressed as means ± SEM. Statistical analyses were performed by using one-way ANOVA followed by Tukey’s test for the comparison of multiple treatment groups. In all comparisons, p values < 0.05 were considered statistically significant.

### Ethical approval

This article does not contain any studies related to human participants or animals.

## Results

### Construction of constitutive expression plasmid pSHY211

As shown in Fig. 1, after the DNA-binding products of TFH1 and TFH2 were transformed into *E. coli* DH5α, a fluorescent clone was screened on the kanamycin-resistant LB medium plate, and the constitutive expression plasmid pSHY211 was successfully obtained. The profile of pSHY211 was shown in Supplementary Figure S2.

### Sequence analysis of XynGMQA

**Figure 2:**
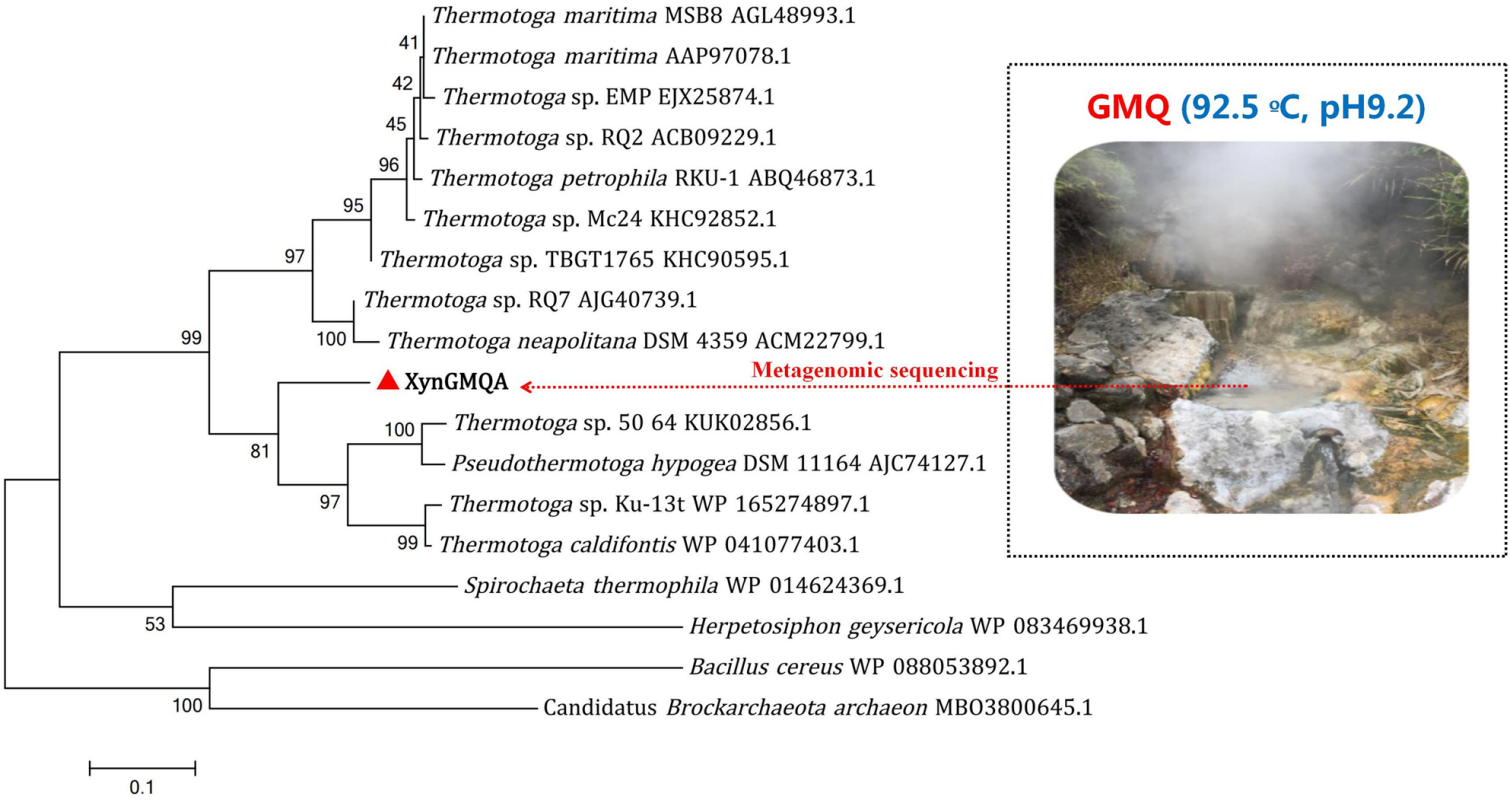
Phylogenetic dendrogram obtained by maximum likelihood analysis based on amino acid sequences showing the phylogenetic position of XynGMQA with related xylanase. Bootstrap values (expressed as a percentage of 1000 replications) are given at nodes.

DNA samples obtained from the sandy soil of Gumingquan hot spring (92.5 °C, pH 9.2) were subjected to sequencing, which generated a total of 4.8 Gbps with 12, 083 contigs of > 500 bp in length (data not shown). A similar search for beta-xylanase in contigs revealed a new candidate xylanase gene sequence, which was denominated *xyngmqa*. Nucleotide sequence analysis of the complete XynGMQA gene revealed 1053 bp ORF encoding a xylanase protein of 350 amino acid residues. No signal peptide sequence was found. The theoretically calculated molecular size and theoretical pI of the recombinant XynGMQA were 42.73 kDa and 6.17, respectively. The amino acid sequence of XynGMQA showed 82.70%, 81.23%, 79.81%, and 80.43% identity to endo-1,4-beta-xylanase (NCBI accession NO.: WP_231848539.1) from *Thermotoga caldifontis*, endo-1,4-beta-xylanase (NCBI accession NO.: WP_241240576.1) from *Thermotoga* sp. Ku-13t, endo-1,4-beta-xylanase (GenBank accession NO.: KUK02856.1) from *Thermotoga* sp. 50_64 and endo-1,4-beta-xylanase (NCBI accession NO.: WP_031505017.1) from *Pseudothermotoga hypogea*. A phylogenetic analysis of protein sequences revealed XynGMQA clustered with other endo-1,4-beta-xylanases from *Thermotoga* and *Pseudothermotoga* (Fig. 2).

### Cloning of XynGMQA gene in E. coli DH5α

**Figure 3:**
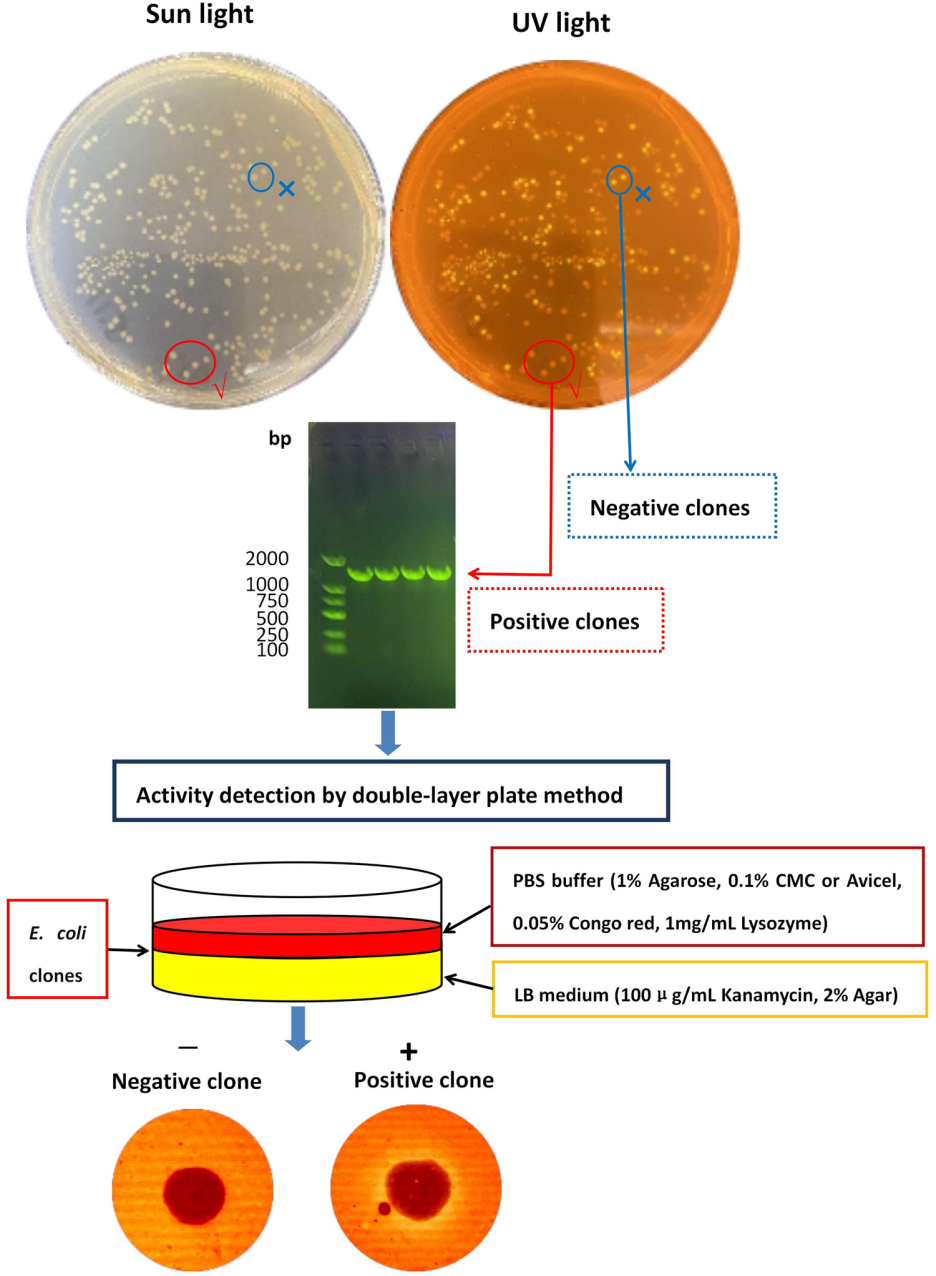
Screening of xylanase active clones (*E. coli* DH5α/*pSHY211-XynGMQA*) by double-layer plate method in *E. coli* DH5α. Complete agarose gel and plate images were shown in Supplementary Figure S4 and Supplementary Figure S5, respectively.

As shown in Fig. 3, the XynGMQA gene linked with pSHY211 was successfully cloned into *E. coli* DH5α with N-His as a fusion protein with His-tag, which was further confirmed by PCR and sequencing. Here, the enhanced green fluorescent protein (EGFP) gene was replaced by the target gene, and positive recombinants could be quickly identified according to the presence (Negative clones, *E. coli* DH5α/*pSHY211*) or absence (Positive clones, *E. coli* DH5α/*pSHY211-XynGMQA*) of green fluorescence in the colonies under ultraviolet (UV) light. Further colony PCR and sequencing also showed that the four non-fluorescent colonies were all positive clones. Xylanase activity of positive clones was detected by DLP.

### Heterologous expression and purification of XynGMQA

**Figure 4:**
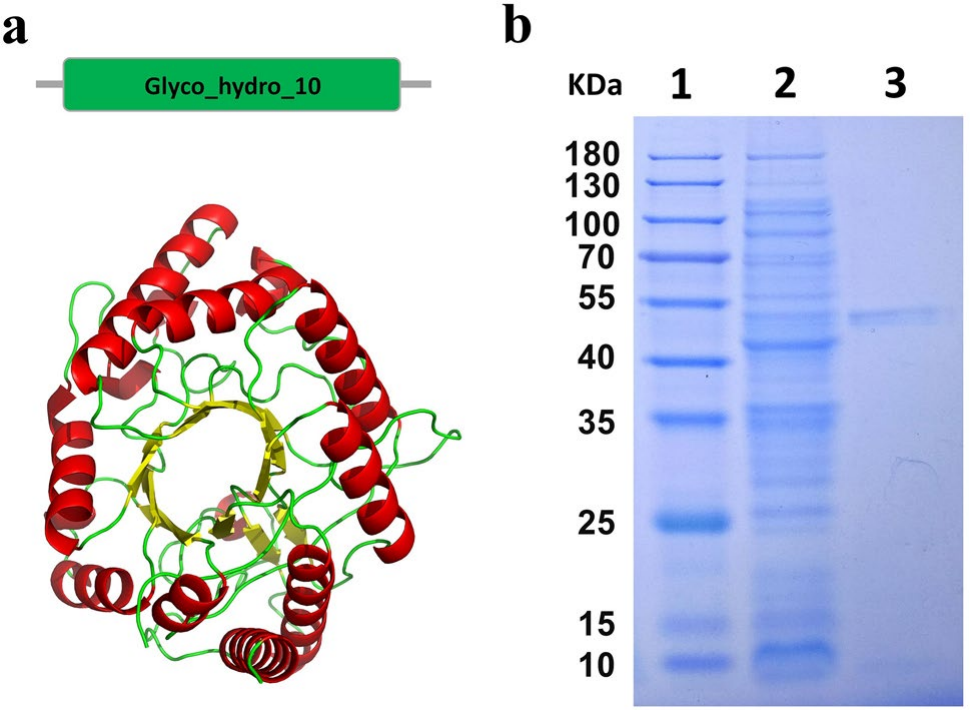
The tertiary structure (**a**) and SDS-PAGE analysis of XynGMQA (**b**). Lane 1, protein molecular weight marker, mass indicated on the left; lane 2, total protein of *E. coli* DH5α/*pSHY211-XynGMQA*; lane 3, purified XynGMQA. The recombinant XynGMQA were 42.73 kDa. Complete SDS-PAGE image was shown in Supplementary Figure S6.

As observed in Fig. 4, XynGMQA has a catalytic domain, which was similar to the GH10 family domain of endo-β-1, 4-xylanase. Multiple sequence alignments of XynGMQA with the closest structure-resolved xylanase were performed (Supplementary Figure S3). Two putative catalytic residues (E155 and E262) were found in XynGMQA. The recombinant xylanase (XynGMQA) was successfully expressed and purified. The purified protein showed a single band of ~ 43 kDa against the protein marker on 10% SDS-PAGE. This is consistent with the theoretical value of the protein. The protein yield of purified recombinant XynGMQA was about 28 mg per liter of LB medium.

### Effect of temperature and pH on XynGMQA

**Figure 5:**
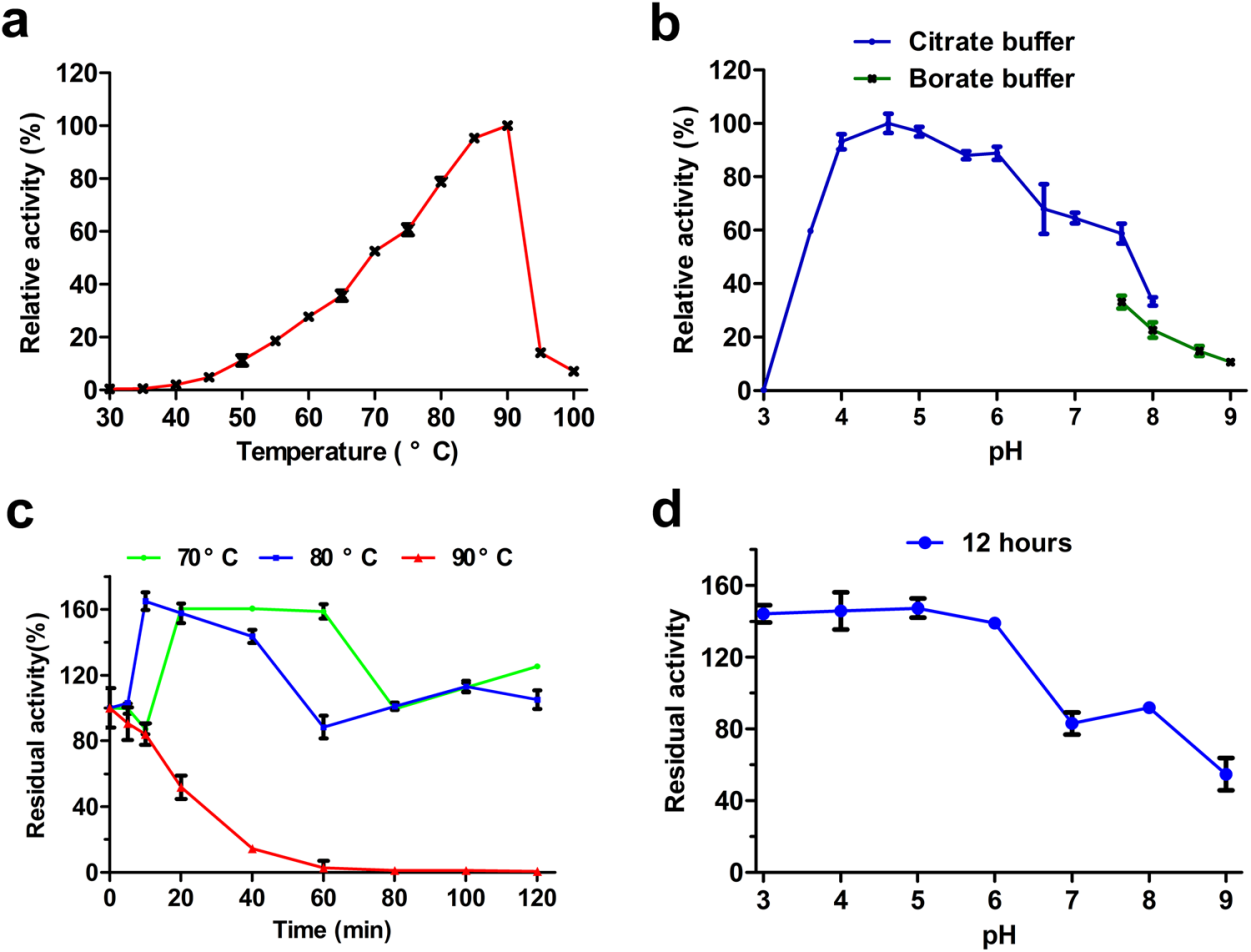
Effects of temperature and pH on the activity and stability of the recombinant XynGMQA. (**a**) Temperature effect on the activity of XynGMQA. (**b**) pH effect on the activity of XynGMQA. (**c**) The effect of temperature on stability at different temperatures (70, 80, and 90 °C) for 0, 20, 40, 60, 80, 100, and 120 min. (**d**) The effect of pH on stability. The primary activity was taken as 100%. Each value in the Figure represents the mean ± SD (n = 3). 100% = 3.4 ± 0.3 U/mg.

The optimal reaction temperature for XynGMQA activity was 90 °C, and over 50% of the maximal activity was observed at 70 to 90 °C (Fig. 5a). The optimal pH for XynGMQA activity was pH 4.6, and over 80% of maximal activity was maintained between pH 4.0 and 6.0 (Fig. 5b). Thermostability analysis showed that XynGMQA retained 80% activity after heat treatment at 80 °C for 2 h and its half-life at 90 °C was about 19 min (Fig. 5c). Interestingly, its enzyme activity was enhanced by high temperatures (70 and 80 °C) and low pH (3.0–6.0). About 150% enzyme activity was detected after incubation at 70 °C for 20 to 60 min or 80 °C for 10 to 40 min (Fig. 5c), and more than 140% enzyme activity after incubation in pH 3.0 to 6.0 for 12 h (Fig. 5d).

### Effect of metal ions and chemical agents on XynGMQA

As shown in Supplementary Table S2, XynGMQA activity was activated by Fe^3+^ (115.2 ± 0.5%), slightly deactivated by K^+^, Mg^2+^, Fe^2+^, Ca^2+^, Ni^2+^, Ba^2+^, Pb^2+^, Zn^2+^, Al^3+^, and strongly inhibited by Co^2+^ and Ag^2+^ at 10 mM metal ions. It almost lost all activity in the presence of 10 mM Cu^2+^ or Mn^2+^. Tween 20, Tween 80, and PFMS had no effect on xylanase activity at 1%, but its activity was slightly inhibited by EDTA and Tween 60. XynGMQA was highly inhibited by 1% SDS and 10% methyl alcohol. While, it still retained over 60% of maximal activity in the presence of 10% ionic liquid and isopropyl alcohol, and over 45% of maximal activity in the presence of 10% ethyl alcohol and 1% DTT. It was suggested that Co^2+^, Ag^2+^, Cu^2+^, Mn^2+^, SDS, and methyl alcohol should be avoided during the application of XynGMQA.

### Substrate specificity and kinetic analysis of XynGMQA

The substrate specificity of XynGMQA was shown in Table 1. It exhibited activities for beechwood xylan (3.4 ± 0.3 U/mg), oat xylan (0.3 ± 0.05 U/mg), and avicel (0.35 ± 0.04 U/mg), but no activity for 1,3;1,4-beta-glucan, CMC, soluble starch and pNPX. The Km, Vmax, and Kcat of recombinant XynGMQA for beechwood xylan were 2.1 ± 0.2 mg/mL, 6.6 ± 0.3 μmol/min/mg and 4.5 ± 0.2 S^-1^, respectively (Table 2).

**Table 1:** Substrate specificities of XynGMQA.

Substrate	Special activity (U/mg)
Beechwood xylan	3.4 ± 0.3
Oat xylan	0.3 ± 0.05
Avicel	0.35 ± 0.04
Beta-1,3;1,4-glucan	0
CMC	0
Starch	0
pNPX	0

**Table 2:** Kinetic parameters of XynGMQA.

Substrate	Vmax (µmol/min/mg)	Km	Kcat (S-1)	Kcat/Km
Beechwood xylan	6.6 ± 0.3	2.1 ± 0.2	4.5 ± 0.2	2.1 ± 0.1

### TLC analysis of hydrolysis products

**Figure 6:**
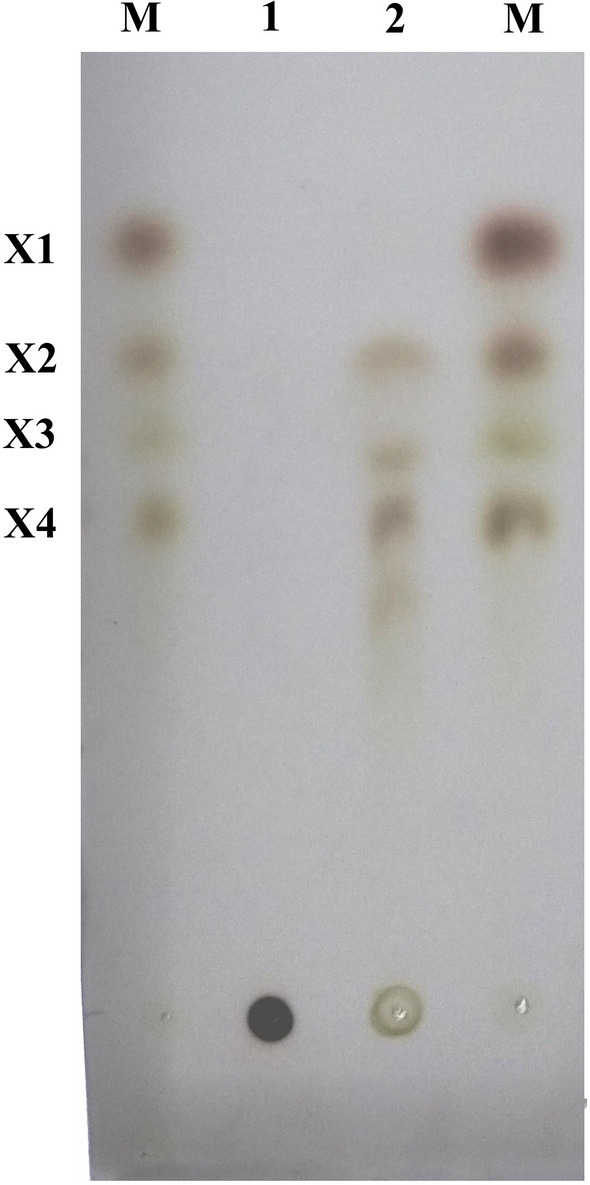
Thin-Layer Chromatography (TLC) of hydrolyzation products of xylooligosaccharides by XynGMQA. Lane 1, standards: X1 (xylose), X2 (xylobiose), X3 (xylotriose), and X4 (xylotetraose); lane 2, beechwood xylan without enzyme; lane 3, beechwood xylan hydrolysis by purified XynGMQA.

As shown in Fig. 6, hydrolytic products of beechwood xylan with XynGMQA were analyzed by TLC. The results showed that the main products of XynGMQA were X2, X3, and X4.

## Discussion

### The constitutive expression plasmid pSHY211

In this study, a constitutive expression plasmid (denominated pSHY211) was constructed for rapid screening of clones. In other words, the EGFP gene was replaced by the target gene, and the non-fluorescent clones were selected directly under UV light (Fig. 3). This plasmid can be used to screen recombinants directly on the culture plate and eliminate unloading clones. Compared with pET28a, the expression of the target gene in *E.coli* DH5α can be achieved by using plasmid pSHY211 in cloning without replacing the host cell^23^. Furthermore, the plasmid pSHY211 was simpler to screen for target genes than the commonly used blue-white selection^24^, without the addition of reagents in the medium, such as X-Gal and IPTG. This effectively saved many experimental steps and time in the process of cloning and expression of functional genes. For example, it can be used in gene cloning to quickly remove false positive clones and evaluate the positive rate in transformants. These indicated that pSHY211 was unaffected by *E. coli* strains and inducers, and may have potential applications for rapid expression and high throughput screening of target genes.

### Double-layer plate (DLP) method

DLP method based on plasmid pSHY211 was established, which can quickly screen active clones. In particular, many functional enzymes related to the degradation of macromolecular substrates, such as proteins, and polysaccharides need to be secreted outside the cell^25,26^. To establish rapid screening of clones on the plate, the signal peptide of functional enzymes must be recognized, transported extracellularly, and removed by *E. coli*^27,28^. However, many studies have shown that these signal peptides from other species are not well recognized by *E. coli*^29,30^. If the functional enzyme has a signal peptide, the DNA fragment of the signal peptide is usually deleted when *E. coli* is used as a host cell for heterologous expression^31^. In this study, the hot spring metagenome-derived xylanase gene (*xyngmqa*), which does not have a signal peptide, may be part of the complete gene sequence. However, as shown in Fig. 3, the xylanase was successfully released into the medium to degrade the xylan by adding appropriate lysozyme in the second plate medium, so that clones with functional genes could be quickly screened. In addition to xylanase screening, this method can also be used for rapid screening of functional enzymes, such as cellulase, pectinase, chitinase, amylase, lipase, etc.^32^, which need to be secreted to the extracellular by replacing substrates and detection methods.

### Sequence and function analysis of XynGMQA

Like other GH10 xylanases, the tertiary structure of XynGMQA shows a (β/α)_8_-barrel structure or TIM-barrel folding^33,34^ (Fig. 4). A phylogenetic analysis of protein sequences revealed XynGMQA clustered with other xylanases from *Thermotoga* and *Pseudothermotoga*. *Thermotoga* spp. and *Pseudothermotoga* spp. are members of the phylum *Thermotogota*, class *Thermotogae*, order *Thermotogales*, and family *Thermotogaceae*^35^. Known habitats of them are mainly high-temperature environments, such as hot springs, oil reservoirs, and thermophilic bioreactors^35,36^. Some studies have shown that xylanases from these two genera also have high thermal stability^37^. In addition, the water temperature of GMQ reached 92.5 °C, which also indicates that XynGMQA may be a thermophilic xylanase. These suggest that thermophilic microorganisms from hot springs are an important potential source of extremely thermophilic xylanases and other enzymes.

### Enzymatic properties of XynGMQA

The optimum reaction temperature of XynGMQA was as high as 90 °C, which was consistent with its origin in the hot spring habitat, indicating that XynGMQA may be involved in the carbon metabolism of hemicellulose in the hot spring. In recent years, metagenomics, as an important technical means of studying uncultured microorganisms and discovering new functional genes^38,39^, provides an opportunity to further study the biological dark matter in hot springs^40^. As shown in Table 3, compared with other environmental metagenomic sources of xylanases, the optimum temperature of xylanases from hot springs was 65–90°C^9,41,42^, that of xylanases from compost was 50–80°C^43,44^, and that of xylanases from intestinal microbes, such as termite gut, cattle rumen, camel rumen, and chicken cecum, was 37–65°C^45–50^.

**Table 3:** Comparison of XynGMQA with xylanases from other environments.

Name	Source	Pfam	Opt. T. (°C)	Opt. pH	Thermostability	References
XynGMQA	Hot spring	GH10	90	4.6	> 80% (80 °C , 2 h), 50% (90 °C , 19 min)	This study
XynDRTY1	Hot spring	GH10	65	6.0	50% (65 °C, 38 min)	^9^
Pm25	Termite gut	GH10	60	4.5–9.0	> 80% (50 °C , 24 h), < 20% (60 °C , 1 h)	^50^
XylR	Nellore cattle rumen	GH10	37	6.0	> 80% (50 °C, 1 h)	^49^
XynM1	Hot spring	GH10	80	7.0	50% (70 °C 1.5 h)	^42^
XylCMS	Camel rumen	GH11	55	6.0	> 40% (55 °C , 50 min)	^48^
XynA3	Hot Spring	GH11	80	6.5	> 70% (70 °C for 24 h)	^41^
PersiXyn1	Camel rumen	GH10	40	8.0	80% (40 °C, 1 h)	^47^
XynAMG1	Chicken cecum	GH10	45	6.0	> 70% (50 °C , 1 h)	^46^
Biof1_09	Compost	GH43	50	4.5	50% (70 °C , 1 h)	^44^
Mxyl	Compost-soil	GH11	80	9.0	50% (80 °C , 2 h; 90 °C, 15 min)	^43^
RuCelA	Yak rumen	GH5	65	7.0	60% (60 °C , 1 h); < 10% (65 °C , 1 h)	^45^

Opt. T. means optimum temperature. Opt. pH means optimum pH.

Although XynGMQA is not high in special activity (3.4 ± 0.3 U/mg), its other enzymatic properties are superior, such as thermophilic and thermotolerant. After incubation at 80 °C for 2 h and 90 °C for 19 min, XynGMQA showed more than 80% and 50% residual activity, respectively. Xylanase from other high-temperature environments, like hot springs and composts, showed higher thermostability than xylanases derived from intestinal microbes (Table 3). For example, XynM1 from a hot spring showed more than 70% residual activity after incubation at 70 °C for 24 h^42^, and Mxyl from compost-soil exhibited 50% residual activity after incubation at 80 °C for 2 h or 90 °C for 15 min^43^. However, xylans of intestinal origin will lose most of their activity at high temperatures, for instance, Pm25 from termite gut lost more than 80% of its relative activity when incubated at 60 °C for 1 h^50^, and RuCelA lost more than 90% of its relative activity when incubated at 65 °C for 1 h^45^. These studies indicate that the typical high-temperature environment should be considered in the process of using metagenomics to mine new thermophilic enzymes.

Xylooligosaccharides have been widely used as oligosaccharide additives in recent years^51^. They consist of 2 ~ 7 xyloses linked by β-1,4 glycosidic bonds. They have the functions of improving immunity, regulating intestinal flora, and improving the quality of livestock and poultry products^52^. XynGMQA hydrolyzed beechwood xylan products were mainly X2, X3, and X4 (Fig. 6), and these xylooligosaccharides may be added to food and feed as prebiotics. As an extremely thermophilic xylanase, XynGMQA is expected to have unique advantages in the production of xylooligosaccharides from lignocellulosic biomass, such as tolerance to industrial high-temperature processes and pollution avoidance^53^. However, its hydrolysates are unknown xylooligosaccharides, and more supporting tests are needed in the future if it is to be used in prebiotic production.

## Conclusion

In summary, a constitutive expression plasmid (denominated pSHY211) and a novel screening method (named DLP method) were constructed for rapid cloning, expression, and identification of xylanase (XynGMQA) from Gumingquan hot spring in Tengchong City, Yunnan Province, southwest China. Detailed enzymatic characterization of XynGMQA showed that it was an extremely thermophilic xylanase. In addition, XynGMQA can hydrolyze xylan to xylooligosaccharides, including xylobiose (X2), xylotriose (X3), and xylotetraose (X4). Overall, in this work, pSHY211 and DLP can be used for rapid heterologous expression and functional gene screening, and XynGMQA may be exploited for biofuel and prebiotic production.

### Supplementary Information

Supplementary Information.

## Data Availability

Original contributions presented in the study are included in the article/Supplementary Materials. The nucleotide sequence of the XynGMQA gene was submitted to GenBank (https://www.ncbi.nlm.nih.gov/nuccore/MW131969). Further inquiries can be directed to the corresponding authors.

## References

[1] Pauly M, Keegstra K (2008). Cell-wall carbohydrates and their modification as a resource for biofuels. Plant J..

[2] Takkellapati S, Li T, Gonzalez MA (2018). An overview of biorefinery-derived platform chemicals from a cellulose and hemicellulose biorefinery. Clean Technol. Environ. Policy.

[3] Alokika, Singh B (2019). Production, characteristics, and biotechnological applications of microbial xylanases. Appl. Microbiol. Biotechnol..

[4] Soni M, Mathur C, Soni A, Solanki MK, Kamboj DV (2020). Xylanase in waste management and its industrial applications. Waste Energy: Prospect. Appl..

[5] He J, Tang F, Chen D, Yu B, Luo Y, Zheng P (2019). Design, expression and functional characterization of a thermostable xylanase from *Trichoderma reesei*. PLoS ONE.

[6] Yin YR, Hu QW, Xian WD, Zhang F, Zhou EM, Ming H (2017). Characterization of a neutral recombinant xylanase from *Thermoactinospora rubra* YIM 77501T. Antonie Van Leeuwenhoek.

[7] Bergquist PL, Gibbs MD, Morris DD, Thompson DR, Daniel RM (2001). Hyperthermophilic xylanases. Method. Enzymol..

[8] Ellis JT, Magnuson TS (2012). Thermostable and alkalistable xylanases produced by the thermophilic bacterium *Anoxybacillus flavithermus* TWXYL3. Isrn Microbiol..

[9] Yin YR, Li L, Yang RF (2022). Characterization of a metagenome-derived thermostable xylanase from Tengchong hot spring. Biomass Conv. Bioref..

[10] Uchiyama T, Miyazaki K (2009). Functional metagenomics for enzyme discovery: Challenges to efficient screening. Curr. Opin. Biotechnol..

[11] Tansirichaiya S, Hutton W, Roberts AP (2023). Functional and sequence-specific screening protocols for the detection of novel antimicrobial resistance genes in metagenomic DNA. Metagenomics Part Method. Mol. Biol..

[12] Montella S, Amore A, Faraco V (2015). Metagenomics for the development of new biocatalysts to advance lignocellulose saccharification for bioeconomic development. Crit. Rev. Biotechnol..

[13] Ruller R, Rosa JC, Faca VM, Greene LJ, Ward RJ (2006). Efficient constitutive expression of *Bacillus subtilis* xylanase a in *Escherichia coli* DH5α under the control of the *Bacillus Bsxa* promoter. Biotechnol. Appl. Biochem..

[14] Zerbino DR, Birney E (2008). Velvet: Algorithms for de novo short read assembly using de Bruijn graphs. Genome Res..

[15] Nakaya A, Katayama T, Itoh M, Hiranuka K, Kawashima S, Moriya Y, Goto S (2012). KEGG OC: A large-scale automatic construction of taxonomy-based ortholog clusters. Nucleic Acids Res..

[16] Tatusov RL (2001). The COG database: New developments in phylogenetic classification of proteins from complete genomes. Nucleic Acids Res..

[17] Finn RD, Tate J, Mistry J, Coggill PC, Sammut SJ, Hotz HR, Bateman A (2007). The Pfam protein families database. Nucleic Acids Res..

[18] Thompson JD, Gibson TJ, Plewniak F, Jeanmougin F, Higgins DG (1997). The CLUSTAL_X windows interface: Flexible strategies for multiple sequence alignment aided by quality analysis tools. Nucleic Acids Res..

[19] Kumar S, Stecher G, Tamura K (2016). MEGA7: Molecular evolutionary genetics analysis version 7.0 for bigger datasets. Mol. Biol. Evol..

[20] Šali A, Blundell TL (1993). Comparative protein modelling by satisfaction of spatial restraints. J. Mol. Biol..

[21] Larkin MA, Blackshields G, Brown NP, Chenna R, McGettigan PA, McWilliam H, Valentin F, Wallace IM, Wilm A, Lopez R, Thompson JD, Gibson TJ, Higgins DG (2007). Clustal W and Clustal X version 2.0. Bioinformatics.

[22] Miller GL (1959). Use of dinitrosalicylic acid reagent for determination of reducing sugar. Anal. Biochem..

[23] Yin YR, Meng ZH, Hu QW (2017). The hybrid strategy of *Thermoactinospora rubra* YIM 77501T for utilizing cellulose as a carbon source at different temperatures. Front. Microbiol..

[24] Faan YW, Yu M, Tsang J (2007). Blue-white selection of regulatory genes that affect the expression of dehalogenase IVa of *Burkholderia cepacia* MBA4. Appl. Microbiol. Biotechnol..

[25] Esquivel J, Voget CE (2004). Purification and partial characterization of an acidic polygalacturonase from *Aspergillus kawachii*. J. Biotechnol..

[26] Mahalik S, Mohapatra D, Kumar D (2018). Cellulase production in lysinibacillus sp isolated from the estuaries of odisha. Biosci. Biotechnol. Res. Commun..

[27] Suzuki T, Ichihara S, Mizushima S (1988). Purification and characterization of a signal peptide, a product of protein secretion across the cytoplasmic membrane of *Escherichia coli*. J. Biochem..

[28] Grady LM, Michtavy J, Oliver DB (2012). Characterization of the *Escherichia coli* SecA signal peptide-binding site. J. Bacteriol..

[29] Hewinson RG, Harris DP, Whelan A, Russell WP (1996). Secretion of the mycobacterial 19-kilodalton protein by *Escherichia coli*, a novel method for the purification of recombinant mycobacterial antigens. Clin. Diagn. Lab. Immunol..

[30] Pournejati R, Karbalaei-Heidari HR, Budisa N (2014). Secretion of recombinant archeal lipase mediated by svp2 signal peptide in *Escherichia coli* and its optimization by response surface methodology. Protein Expr. Purif..

[31] Yin YR, Li T, Sang P, Yang RF (2022). Characterization of an alkali-tolerant, thermostable, and multifunctional GH5 family endoglucanase from *Thermoactinospora rubra* YIM 77501T for prebiotic production. Biomass Conv. Bioref..

[32] Patel JN, Parmar FA, Upasani VN (2021). Isolation and characterization of pathogens causing disease in pomegranate (*Punica granatum* l.), India. Int. J. Innov. Res. Sci. Eng. Technol..

[33] Leggio LL, Kalogiannis S, And M, Pickersgill RW (1999). High resolution structure and sequence of T. aurantiacus xylanase I: implications for the evolution of thermostability in family 10 xylanases and enzymes with (beta) alpha-barrel architecture. Proteins: Struct. Funct., Bioinform..

[34] Niderhaus C, Garrido M, Insani M, Campos E, Wirth S (2018). Heterologous production and characterization of a thermostable GH10 family endo-xylanase from *Pycnoporus sanguineus* BAFC 2126. Process Biochem..

[35] Farrell, A., Nesb, C. L., Zhaxybayeva, O., L'Haridon, S. Pseudothermotoga. *American Cancer Society* (2021).

[36] Roumagnac M, Pradel N, Bartoli M, Garel M, Jones A, Armougom F (2020). Responses to the hydrostatic pressure of surface and subsurface strains of Pseudothermotoga elfii revealing the piezophilic nature of the strain originating from an oil-producing well. Front. Microbiol..

[37] Mamo G, Kasture S, Faryar R, Hashim S, Hatti-Kaul R (2010). Surfactants from xylan: production of n-octyl xylosides using a highly thermostable xylanase from thermotoga neapolitana. Process Biochem..

[38] Hakeem KR, Bilal T, Malik B (2018). Metagenomic analysis of uncultured microorganisms and their enzymatic attributes. J. Microbiol. Methods.

[39] Alawiye TT, Babalola OO (2021). Metagenomic insight into the community structure and functional genes in the sunflower rhizosphere microbiome. Agriculture.

[40] Stone R (2011). Biological dark matter exerts irresistible pull in Yunnan. Science.

[41] Knapik K, Becerra M, María-Isabel GS (2019). Microbial diversity analysis and screening for novel xylanase enzymes from the sediment of the Lobios Hot Spring in Spain. Sci. Rep..

[42] Joshi N, Sharma M, Singh SP (2020). Characterization of a novel xylanase from an extreme temperature hot spring metagenome for xylooligosaccharide production. Appl. Microbiol. Biotechnol..

[43] Verma D, Kawarabayasi Y, Miyazaki K (2013). Cloning, expression and characteristics of a novel alkalistable and thermostable xylanase encoding gene (Mxyl) retrieved from compost-soil metagenome. PLoS ONE.

[44] Ritthironk SL, Boonmee A (2014). Newly derived GH43 gene from compost metagenome showing dual xylanase and cellulase activities. Folia Microbiol..

[45] Chang L, Ding M, Bao L (2011). Characterization of a bifunctional xylanase/endoglucanase from yak rumen microorganisms. Appl. Microbiol. Biotechnol..

[46] Al-Darkazali H, Meevootisom V, Isarangkul D, Wiyakrutta S (2017). Gene expression and molecular characterization of a xylanase from chicken cecum metagenome. Int. J. Microbiol..

[47] Ariaeenejad S, Hosseini E, Maleki M, Kavousi K, Moosavi-Movahedi AA, Salekdeh GH (2019). Identification and characterization of a novel thermostable xylanase from camel rumen metagenome. Int. J. Biol. Macromol..

[48] Ghadikolaei KK, Sangachini ED, Vahdatirad V (2019). An extreme halophilic xylanase from camel rumen metagenome with elevated catalytic activity in high salt concentrations. AMB Expr..

[49] Gabriella CP, Eliana GDML, Natália SML, Joo MP (2021). Characterization of a new bifunctional endo-1,4-β-xylanase/esterase found in the rumen metagenome. Sci. Rep..

[50] Wu H, Ioannou E, Henrissat B (2021). Multimodularity of a GH10 xylanase found in the termite gut metagenome. Am. Soc. Microbiol..

[51] Monteiro C, Vila PF, Pereira M, Pereira GN, Poletto P (2021). Hydrothermal treatment on depolymerization of hemicellulose of mango seed shell for the production of xylooligosaccharides. Carbohydrate Polym..

[52] Lvarez C, González A, Ballesteros I, Negro MJ (2021). Production of xylooligosaccharides, bioethanol, and lignin from structural components of barley straw pretreated with a steam explosion. Bioresour. Technol..

[53] Heinen PR, Pereira MG, Rechia C, Almeida PZ, Monteiro L, Pasin TM (2017). Immobilized endo-xylanase of aspergillus tamarii kita: An interesting biological tool for production of xylooligosaccharides at high temperatures. Process Biochem..

