# Supplementary material for: Characterization of a GH10 extremely thermophilic xylanase from the metagenome of hot spring for prebiotic production
Source: Sci Rep. 2023 Sep 25;13:16053. doi: 10.1038/s41598-023-42920-6 (PMC10520001; doi:10.1038/s41598-023-42920-6)
Supplement: Supplementary file 1 — Supplementary Information. [file 41598_2023_42920_MOESM1_ESM.pdf]

SUPPLEMENTARY MATERIAL

Supplementary Table S1 Primers design of pSHY211.

Primers	**Sequences (5′—3′)**
TFH-F1	CCCTATAGTGAGTCGTATTAATTTCGCGGGATCG
TFH-R1	GAGCTCCGTCGAC**AAGCTT**GCGGC
TFH-F2	GGGGTACCTAGCGTGGTATTAT
TFH-R2	ATC**AAGCTT**TTACTTGTACAGCTCG

The underlined DNA sequnces are Hind III restriction enzyme recognizes site (AAGCTT). The DNA seqences of TFH1 and TFH2 are as following below.

> TFH1 (The underlined DNA sequnce is Hind III restriction enzyme recognizes site.)

GAGCTCCGTCGAC**AAGCTT**GCGGCCGCACTCGAGCACCACCACCACCACCACTGAGATCC
GGCTGCTAACAAAGCCCGAAAGGAAGCTGAGTTGGCTGCTGCCACCGCTGAGCAATAACT
AGCATAACCCCTTGGGGCCTCTAAACGGGTCTTGAGGGGTTTTTTGCTGAAAGGAGGAACT
ATATCCGGATTGGCGAATGGGACGCGCCCTGTAGCGGCGCATTAAGCGCGGCGGGTGTGGT
GGTTACGCGCAGCGTGACCGCTACACTTGCCAGCGCCCTAGCGCCCGCTCCTTTCGCTTTCT
TCCCTTCCTTTCTCGCCACGTTCGCCGGCTTTCCCCGTCAAGCTCTAAATCGGGGGCTCCCT
TTAGGGTTCCGATTTAGTGCTTTACGGCACCTCGACCCCAAAAAACTTGATTAGGGTGATGG
TTCACGTAGTGGGCCATCGCCCTGATAGACGGTTTTTCGCCCTTTGACGTTGGAGTCCACGT
TCTTTAATAGTGGACTCTTGTTCCAAACTGGAACAACACTCAACCCTATCTCGGTCTATTCTT
TTGATTTATAAGGGATTTTGCCGATTTCGGCCTATTGGTTAAAAAATGAGCTGATTTAACAAA
AATTTAACGCGAATTTTAACAAAATATTAACGTTTACAATTTCAGGTGGCACTTTTCGGGGA
AATGTGCGCGGAACCCCTATTTGTTTATTTTTCTAAATACATTCAAATATGTATCCGCTCATGA
ATTAATTCTTAGAAAAACTCATCGAGCATCAAATGAAACTGCAATTTATTCATATCAGGATTA
TCAATACCATATTTTTGAAAAAGCCGTTTCTGTAATGAAGGAGAAAACTCACCGAGGCAGT
TCCATAGGATGGCAAGATCCTGGTATCGGTCTGCGATTCCGACTCGTCCAACATCAATACAA
CCTATTAATTTCCCCTCGTCAAAAATAAGGTTATCAAGTGAGAAATCACCATGAGTGACGAC
TGAATCCGGTGAGAATGGCAAAAGTTTATGCATTTCTTTCCAGACTTGTTCAACAGGCCAGC
CATTACGCTCGTCATCAAAATCACTCGCATCAACCAAACCGTTATTCATTCGTGATTGCGCCT
GAGCGAGACGAAATACGCGATCGCTGTTAAAAGGACAATTACAAACAGGAATCGAATGCA
ACCGGCGCAGGAACACTGCCAGCGCATCAACAATATTTTCACCTGAATCAGGATATTCTTCT
AATACCTGGAATGCTGTTTTCCCGGGGATCGCAGTGGTGAGTAACCATGCATCATCAGGAGT
ACGGATAAAATGCTTGATGGTCGGAAGAGGCATAAATTCCGTCAGCCAGTTTAGTCTGACC
ATCTCATCTGTAACATCATTGGCAACGCTACCTTTGCCATGTTTCAGAAACAACTCTGGCGC
ATCGGGCTTCCCATACAATCGATAGATTGTCGCACCTGATTGCCCGACATTATCGCGAGCCCA
TTTATACCCATATAAATCAGCATCCATGTTGGAATTTAATCGCGGCCTAGAGCAAGACGTTTC
CCGTTGAATATGGCTCATAACACCCCTTGTATTACTGTTTATGTAAGCAGACAGTTTTATTGT
TCATGACCAAAATCCCTTAACGTGAGTTTTCGTTCCACTGAGCGTCAGACCCCGTAGAAAA
GATCAAAGGATCTTCTTGAGATCCTTTTTTTCTGCGCGTAATCTGCTGCTTGCAAACAAAAA
AACCACCGCTACCAGCGGTGGTTTGTTTGCCGGATCAAGAGCTACCAACTCTTTTTCCGAA

GGTAACTGGCTTCAGCAGAGCGCAGATACCAAATACTGTCCTTCTAGTGTAGCCGTAGTTAG
GCCACCACTTCAAGAACTCTGTAGCACCGCCTACATACCTCGCTCTGCTAATCCTGTTACCA
GTGGCTGCTGCCAGTGGCGATAAGTCGTGTCTTACCGGGTTGGACTCAAGACGATAGTTAC
CGGATAAGGCGCAGCGGTCGGGCTGAACGGGGGGTTCGTGCACACAGCCCAGCTTGGAGC
GAACGACCTACACCGAACTGAGATACCTACAGCGTGAGCTATGAGAAAGCGCCACGCTTCC
CGAAGGGAGAAAGGCGGACAGGTATCCGGTAAGCGGCAGGGTCGGAACAGGAGAGCGCA
CGAGGGAGCTTCCAGGGGGAAACGCCTGGTATCTTTATAGTCCTGTCGGGTTTCGCCACCT
CTGACTTGAGCGTCGATTTTTGTGATGCTCGTCAGGGGGGCGGAGCCTATGGAAAAACGCC
AGCAACGCGGCCTTTTTACGGTTCCTGGCCTTTTGCTGGCCTTTTGCTCACATGTTCTTTCCT
GCGTTATCCCCTGATTCTGTGGATAACCGTATTACCGCCTTTGAGTGAGCTGATACCGCTCGC
CGCAGCCGAACGACCGAGCGCAGCGAGTCAGTGAGCGAGGAAGCGGAAGAGCGCCTGAT
GCGGTATTTTCTCCTTACGCATCTGTGCGGTATTTCACACCGCATATATGGTGCACTCTCAGT
ACAATCTGCTCTGATGCCGCATAGTTAAGCCAGTATACACTCCGCTATCGCTACGTGACTGG
GTCATGGCTGCGCCCCGACACCCGCCAACACCCGCTGACGCGCCCTGACGGGCTTGTCTGC
TCCCGGCATCCGCTTACAGACAAGCTGTGACCGTCTCCGGGAGCTGCATGTGTCAGAGGTT
TTCACCGTCATCACCGAAACGCGCGAGGCAGCTGCGGTAAAGCTCATCAGCGTGGTCGTG
AAGCGATTCACAGATGTCTGCCTGTTCATCCGCGTCCAGCTCGTTGAGTTTCTCCAGAAGC
GTTAATGTCTGGCTTCTGATAAAGCGGGCCATGTTAAGGGCGGTTTTTTCCTGTTTGGTCAC
TGATGCCTCCGTGTAAGGGGGATTTCTGTTCATGGGGGTAATGATACCGATGAAACGAGAG
AGGATGCTCACGATACGGGTTACTGATGATGAACATGCCCGGTTACTGGAACGTTGTGAGG
GTAAACAACTGGCGGTATGGATGCGGCGGGACCAGAGAAAAATCACTCAGGGTCAATGCC
AGCGCTTCGTTAATACAGATGTAGGTGTTCCACAGGGTAGCCAGCAGCATCCTGCGATGCA
GATCCGGAACATAATGGTGCAGGGCGCTGACTTCCGCGTTTCCAGACTTTACGAAACACGG
AAACCGAAGACCATTCATGTTGTTGCTCAGGTCGCAGACGTTTTGCAGCAGCAGTCGCTTC
ACGTTCGCTCGCGTATCGGTGATTCATTCTGCTAACCAGTAAGGCAACCCCGCCAGCCTAGC
CGGGTCCTCAACGACAGGAGCACGATCATGCGCACCCGTGGGGCCGCCATGCCGGCGATAA
TGGCCTGCTTCTCGCCGAAACGTTTGGTGGCGGGACCAGTGACGAAGGCTTGAGCGAGGG
CGTGCAAGATTCCGAATACCGCAAGCGACAGGCCGATCATCGTCGCGCTCCAGCGAAAGC
GGTCCTCGCCGAAAATGACCCAGAGCGCTGCCGGCACCTGTCCTACGAGTTGCATGATAAA
GAAGACAGTCATAAGTGCGGCGACGATAGTCATGCCCCGCGCCCACCGGAAGGAGCTGAC
TGGGTTGAAGGCTCTCAAGGGCATCGGTCGAGATCCCGGTGCCTAATGAGTGAGCTAACTT
ACATTAATTGCGTTGCGCTCACTGCCCGCTTTCCAGTCGGGAAACCTGTCGTGCCAGCTGCA
TTAATGAATCGGCCAACGCGCGGGGAGAGGCGGTTTGCGTATTGGGCGCCAGGGTGGTTTT
TCTTTTCACCAGTGAGACGGGCAACAGCTGATTGCCCTTCACCGCCTGGCCCTGAGAGAGT
TGCAGCAAGCGGTCCACGCTGGTTTGCCCCAGCAGGCGAAAATCCTGTTTGATGGTGGTTA
ACGGCGGGATATAACATGAGCTGTCTTCGGTATCGTCGTATCCCACTACCGAGATATCCGCA
CCAACGCGCAGCCCGGACTCGGTAATGGCGCGCATTGCGCCCAGCGCCATCTGATCGTTGG
CAACCAGCATCGCAGTGGGAACGATGCCCTCATTCAGCATTTGCATGGTTTGTTGAAAACC
GGACATGGCACTCCAGTCGCCTTCCCGTTCCGCTATCGGCTGAATTTGATTGCGAGTGAGAT
ATTTATGCCAGCCAGCCAGACGCAGACGCGCCGAGACAGAACTTAATGGGCCCGCTAACA
GCGCGATTTGCTGGTGACCCAATGCGACCAGATGCTCCACGCCCAGTCGCGTACCGTCTTC
ATGGGAGAAAATAATACTGTTGATGGGTGTCTGGTCAGAGACATCAAGAAATAACGCCGGA
ACATTAGTGCAGGCAGCTTCCACAGCAATGGCATCCTGGTCATCCAGCGGATAGTTAATGAT
CAGCCCACTGACGCGTTGCGCGAGAAGATTGTGCACCGCCGCTTTACAGGCTTCGACGCCG

CTTCGTTCTACCATCGACACCACCACGCTGGCACCCAGTTGATCGGCGCGAGATTTAATCGC
CGCGACAATTTGCGACGGCGCGTGCAGGGCCAGACTGGAGGTGGCAACGCCAATCAGCAA
CGACTGTTTGCCCGCCAGTTGTTGTGCCACGCGGTTGGGAATGTAATTCAGCTCCGCCATCG
CCGCTTCCACTTTTTCCCGCGTTTTCGCAGAAACGTGGCTGGCCTGGTTCACCACGCGGGA
AACGGTCTGATAAGAGACACCGGCATACTCTGCGACATCGTATAACGTTACTGGTTTCACAT
TCACCACCCTGAATTGACTCTCTTCCGGGCGCTATCATGCCATACCGCGAAAGGTTTTGCGC
CATTCGATGGTGTCCGGGATCTCGACGCTCTCCCTTATGCGACTCCTGCATTAGGAAGCAGC
CCAGTAGTAGGTTGAGGCCGTTGAGCACCGCCGCCGCAAGGAATGGTGCATGCAAGGAGA
TGGCGCCCAACAGTCCCCCGGCCACGGGGCCTGCCACCATACCCACGCCGAAACAAGCGC
TCATGAGCCCGAAGTGGCGAGCCCGATCTTCCCCATCGGTGATGTCGGCGATATAGGCGCC
AGCAACCGCACCTGTGGCGCCGGTGATGCCGGCCACGATGCGTCCGGCGTAGAGGATCGA
GATCTCGATCCCGCGAAATTAATACGACTCACTATAGGG

>TFH2 (The blue DNA sequence is promoter of GH11 endoxylanase gene from *Bacillus subtilis* AQ1. The underlined DNA sequnce are EcoR Ⅰ, BamH Ⅰ and Hind Ⅲ restriction enzyme recognizes sites, respectively. The green DNA sequence is EGFP gene.)

GGGGTACCTAGCGTGGTATTATACTGAAGGGGACGATCAAAAGCGTTGGCGTTCGTTA
AATATTTACGAGTGCTGCCTCATGTCAAAGTCAGAAAAAATAGTATAGGAGGTAACATA
TG**GGATCC**GAACATCATCATCATCATCAT**GAATTC**ATGGTGAGCAAGGGCGAGGAGCTGTT
CACCGGGGTGGTGCCCATCCTGGTCGAGCTGGACGGCGACGTAAACGGCCACAAGTTCAG
CGTGTCCGGCGAGGGCGAGGGCGATGCCACCTACGGCAAGCTGACCCTGAAGTTCATCTG
CACCACCGGCAAGCTGCCCGTGCCCTGGCCCACCCTCGTGACCACCCTGACCTACGGCGTG
CAGTGCTTCAGCCGCTACCCCGACCACATGAAGCAGCACGACTTCTTCAAGTCCGCCATGC
CCGAAGGCTACGTCCAGGAGCGCACCATCTTCTTCAAGGACGACGGCAACTACAAGACCC
GCGCCGAGGTGAAGTTCGAGGGCGACACCCTGGTGAACCGCATCGAGCTGAAGGGCATCG
ACTTCAAGGAGGACGGCAACATCCTGGGGCACAAGCTGGAGTACAACTACAACAGCCACA
ACGTCTATATCATGGCCGACAAGCAGAAGAACGGCATCAAGGTGAACTTCAAGATCCGCCA
CAACATCGAGGACGGCAGCGTGCAGCTCGCCGACCACTACCAGCAGAACACCCCCATCGG
CGACGGCCCCGTGCTGCTGCCCGACAACCACTACCTGAGCACCCAGTCCGCCCTGAGCAA
AGACCCCAACGAGAAGCGCGATCACATGGTCCTGCTGGAGTTCGTGACCGCCGCCGGGAT
CACTCTCGGCATGGACGAGCTGTACAAGTAA**AAGCTT**

Supplementary Table S2 Effects of metal ions and reagents on the activity of **XynGMQA.**

**Metal ions and chemical reagents**	**Concentration**	**Relative (%)**
None	0	100±0.5
KCl	10 mM	93.3±0.8
$MgSO_4$	10 mM	69.6±1.9
$FeSO_4$	10 mM	94.7±3.5
$FeCl_3$	10 mM	115.2±1.2
$CaCl_2$	10 mM	80.3±1
$NiSO_4$	10 mM	51.5±3
$CoCl_2$	10 mM	65.4±1.4
$BaCl_2$	10 mM	127.4±1.4
$MnCl_2$	10 mM	0
$AgNO_3$	10 mM	19.8±2.3
$Pb(NO_3)_2$	10 mM	166.8±1.9
$CuSO_4$	10 mM	0
$ZnSO_4$	10 mM	83.7±3.3
$AlCl_3$	10 mM	71.6±0.7
EDTA	1% (w/v)	90±3.9
SDS	1% (w/v)	69.1±4.9
Ionic liquid	10% (v/v)	146.7±4.8
Methyl alcohol	10% (v/v)	18.9±1.5
Ethyl alcohol	10% (v/v)	23.3±1.9
Isopropyl alcohol	1% (w/v)	37±0.5
Tween 20	1% (w/v)	133.2±7.7
Tween 60	1% (w/v)	90.2±2.3
Tween 80	1% (w/v)	141±1.4
DTT	1% (w/v)	0
PFMS	1% (w/v)	75.8±0.9

Statistical analysis was performed using one-way ANOVA followed by Tukey's test for comparison of multiple treatment groups.

Optimized sequence

```
CTGAAAAGTCTGCGTCTGGTTATCCTGGCAGTGGTCCTGTTTTTCGTCCTGAACTTCACC
GTTCAACGTCTGGAAGGCGAAACCCTGCGTACCCTGGCAGAAAAACTGGGCATCTACATC
GGTTACGCGAGCATTAACGATTTTTGGCGCATCACCGACTCCGACAAATACATGGAGGTC
GCGAAACGCGAATTCAACATTCTGACCCCGGAGAACCAGATGAAATGGGATAGCATCCAC
CCGGAAGAAACCCGTTATAACTTCGAACCGGCTGAACGTCACGTACGTTTTGCGCTGGAA
AACGGCATGGTAGTTCACGGTCATACCCTGGTTTGGCATCAACAACTGCCGCCGTGGGTT
TCTGGCAAAAGCACCAAAGAAGAACTGCTGAAAGTCCTGGAGGACCACATCAAAACCGTC
GTTGGCCATTTCAAAGGCCGCGTCAAAATCTGGGATGTTGTCAACGAAGCCGTTAGCGAT
GCAGGTACCTATCGCGATAGCATCTGGTATCGCATCATTGGCCCGGAATACATCGAGAAA
GCGTTTATTTGGGCACACGAAGCAGATCCGGACGCAATCCTGATCTACAACGACTACAAC
ATCGAGACCATCAACCCGAAAAGCAACTTCGTCTACAACATGGTCAAAGAGATGAAAGAG
AAAGGCATCCCGATTCACGGCGTTGGTTTTCAGATGCACATCGACTACAACGGCCTGAAC
TACGAAAGTTTTCGCCAGAACCTGAAACGTTTTGCGGATCTGGGCCTGAAACTGTACATC
ACCGAAATGGACGTTCGCGTTCCGCAAAACGCAACCCCGAAAGATTTCGAAAAACAGGCG
GAGGTCTACCGCAAAATCTTCGAGATCTGCCTGGATAACCCGGCTGTTGAAGCAATTCAG
TTCTGGGGCTTCACCGATAAATACAGCTGGGTTCCGGGCTTTTTCAAAGGCTGGGATCAC
GCGCTGATCTTTGATCGCGATTATAACCCGAAACCGGCGTATTTCGCGATCAAAGAGGTC
CTGGAGAAAAAAGTCATGGAGCGCCAGAAATGA
```

Alignment and translation

```
Wild_type:     1  TTG AAG AGT TTA AGG TTG GTT ATC CTC GCC GTC GTT TTG TTT TTC GTC CTC AAC TTC ACC
Optimized:     1  CTG AAA AGT CTG CGT CTG GTT ATC CTG GCA GTG GTC CTG TTT TTC GTC CTG AAC TTC ACC
                   L   K   S   L   R   L   V   I   L   A   V   V   L   F   F   V   L   N   F   T

Wild_type:    61  GTC CAA AGA TTG GAG GGA GAG ACC TTG AGA ACG CTT GCG GAA AAG CTG GGT ATC TAC ATC
Optimized:    61  GTT CAA CGT CTG GAA GGC GAA ACC CTG CGT ACC CTG GCA GAA AAA CTG GGC ATC TAC ATC
                   V   Q   R   L   E   G   E   T   L   R   T   L   A   E   K   L   G   I   Y   I

Wild_type:   121  GGT TAC GCC TCC ATA AAC GAT TTT TGG AGG ATC ACG GAT TCG GAC AAG TAC ATG GAA GTC
Optimized:   121  GGT TAC GCG AGC ATT AAC GAT TTT TGG CGC ATC ACC GAC TCC GAC AAA TAC ATG GAG GTC
                   G   Y   A   S   I   N   D   F   W   R   I   T   D   S   D   K   Y   M   E   V

Wild_type:   181  GCC AAG AGG GAG TTC AAC ATC CTC ACA CCG GAG AAC CAG ATG AAA TGG GAC AGC ATC CAT
Optimized:   181  GCG AAA CGC GAA TTC AAC ATT CTG ACC CCG GAG AAC CAG ATG AAA TGG GAT AGC ATC CAC
                   A   K   R   E   F   N   I   L   T   P   E   N   Q   M   K   W   D   S   I   H

Wild_type:   241  CCG GAA GAG ACA AGG TAC AAC TTC GAA CCT GCG GAA AGG CAC GTC AGG TTC GCC TTG GAA
Optimized:   241  CCG GAA GAA ACC CGT TAT AAC TTC GAA CCG GCT GAA CGT CAC GTA CGT TTT GCG CTG GAA
                   P   E   E   T   R   Y   N   F   E   P   A   E   R   H   V   R   F   A   L   E

Wild_type:   301  AAT GGG ATG GTT GTC CAC GGA CAC ACG CTG GTT TGG CAC CAG CAA CTC CCT CCT TGG GTG
Optimized:   301  AAC GGC ATG GTA GTT CAC GGT CAT ACC CTG GTT TGG CAT CAA CAA CTG CCG CCG TGG GTT
                   N   G   M   V   V   H   G   H   T   L   V   W   H   Q   Q   L   P   P   W   V

Wild_type:   361  AGC GGG AAG AGC ACG AAG GAA GAA CTC TTG AAA GTT CTC GAA GAC CAT ATA AAG ACG GTT
Optimized:   361  TCT GGC AAA AGC ACC AAA GAA GAA CTG CTG AAA GTC CTG GAG GAC CAC ATC AAA ACC GTC
                   S   G   K   S   T   K   E   E   L   L   K   V   L   E   D   H   I   K   T   V

Wild_type:   421  GTT GGC CAT TTC AAG GGT AGG GTC AAG ATC TGG GAC GTC GTG AAC GAA GCG GTG AGC GAC
Optimized:   421  GTT GGC CAT TTC AAA GGC CGC GTC AAA ATC TGG GAT GTT GTC AAC GAA GCC GTT AGC GAT
                   V   G   H   F   K   G   R   V   K   I   W   D   V   V   N   E   A   V   S   D

Wild_type:   481  GCC GGA ACT TAC AGG GAC AGC ATT TGG TAC AGG ATC ATA GGG CCG GAG TAC ATA GAG AAG
Optimized:   481  GCA GGT ACC TAT CGC GAT AGC ATC TGG TAT CGC ATC ATT GGC CCG GAA TAC ATC GAG AAA
                   A   G   T   Y   R   D   S   I   W   Y   R   I   I   G   P   E   Y   I   E   K

Wild_type:   541  GCG TTC ATC TGG GCC CAC GAG GCT GAT CCT GAC GCG ATC CTC ATC TAC AAC GAC TAC AAC
Optimized:   541  GCG TTT ATT TGG GCA CAC GAA GCA GAT CCG GAC GCA ATC CTG ATC TAC AAC GAC TAC AAC
                   A   F   I   W   A   H   E   A   D   P   D   A   I   L   I   Y   N   D   Y   N

Wild_type:   601  ATC GAA ACG ATC AAC CCC AAG TCC AAC TTC GTC TAC AAC ATG GTG AAA GAG ATG AAG GAG
Optimized:   601  ATC GAG ACC ATC AAC CCG AAA AGC AAC TTC GTC TAC AAC ATG GTC AAA GAG ATG AAA GAG
                   I   E   T   I   N   P   K   S   N   F   V   Y   N   M   V   K   E   M   K   E

Wild_type:   661  AAA GGG ATT CCG ATA CAC GGC GTT GGT TTT CAG ATG CAC ATA GAC TAC AAC GGT TTG AAC
Optimized:   661  AAA GGC ATC CCG ATT CAC GGC GTT GGT TTT CAG ATG CAC ATC GAC TAC AAC GGC CTG AAC
                   K   G   I   P   I   H   G   V   G   F   Q   M   H   I   D   Y   N   G   L   N

Wild_type:   721  TAC GAG AGT TTC AGG CAG AAC CTC AAG AGG TTC GCC GAT CTC GGT CTC AAG CTC TAC ATC
Optimized:   721  TAC GAA AGT TTT CGC CAG AAC CTG AAA CGT TTT GCG GAT CTG GGC CTG AAA CTG TAC ATC
                   Y   E   S   F   R   Q   N   L   K   R   F   A   D   L   G   L   K   L   Y   I

Wild_type:   781  ACG GAG ATG GAC GTG AGA GTG CCT CAG AAC GCG ACG CCG AAA GAC TTC GAA AAA CAA GCC
Optimized:   781  ACC GAA ATG GAC GTT CGC GTT CCG CAA AAC GCA ACC CCG AAA GAT TTC GAA AAA CAG GCG
                   T   E   M   D   V   R   V   P   Q   N   A   T   P   K   D   F   E   K   Q   A

Wild_type:   841  GAA GTC TAC AGG AAG ATC TTC GAG ATA TGC TTG GAC AAC CCG GCG GTC GAG GCC ATA CAG
Optimized:   841  GAG GTC TAC CGC AAA ATC TTC GAG ATC TGC CTG GAT AAC CCG GCT GTT GAA GCA ATT CAG
                   E   V   Y   R   K   I   F   E   I   C   L   D   N   P   A   V   E   A   I   Q

Wild_type:   901  TTT TGG GGT TTC ACG GAC AAG TAC TCG TGG GTC CCG GGG TTC TTC AAA GGT TGG GAC CAC
Optimized:   901  TTC TGG GGC TTC ACC GAT AAA TAC AGC TGG GTT CCG GGC TTT TTC AAA GGC TGG GAT CAC
                   F   W   G   F   T   D   K   Y   S   W   V   P   G   F   F   K   G   W   D   H

Wild_type:   961  GCG CTG ATC TTC GAC AGA GAC TAC AAC CCC AAA CCA GCT TAT TTT GCG ATA AAG GAA GTG
Optimized:   961  GCG CTG ATC TTT GAT CGC GAT TAT AAC CCG AAA CCG GCG TAT TTC GCG ATC AAA GAG GTC
                   A   L   I   F   D   R   D   Y   N   P   K   P   A   Y   F   A   I   K   E   V

Wild_type:  1021  CTG GAG AAA AAG GTG ATG GAG AGG CAG AAA TGA
Optimized:  1021  CTG GAG AAA AAA GTC ATG GAG CGC CAG AAA TGA
                   L   E   K   K   V   M   E   R   Q   K   *
```

Supplementary Figure S1 Gene Optimization of *xyngmqa*. Wild_type represents the original gene sequence of the *xyngmqa* from the metagenomic database. Optimized represents the optimized sequence of the *xyngmqa* based on the codon of *E. coli*.

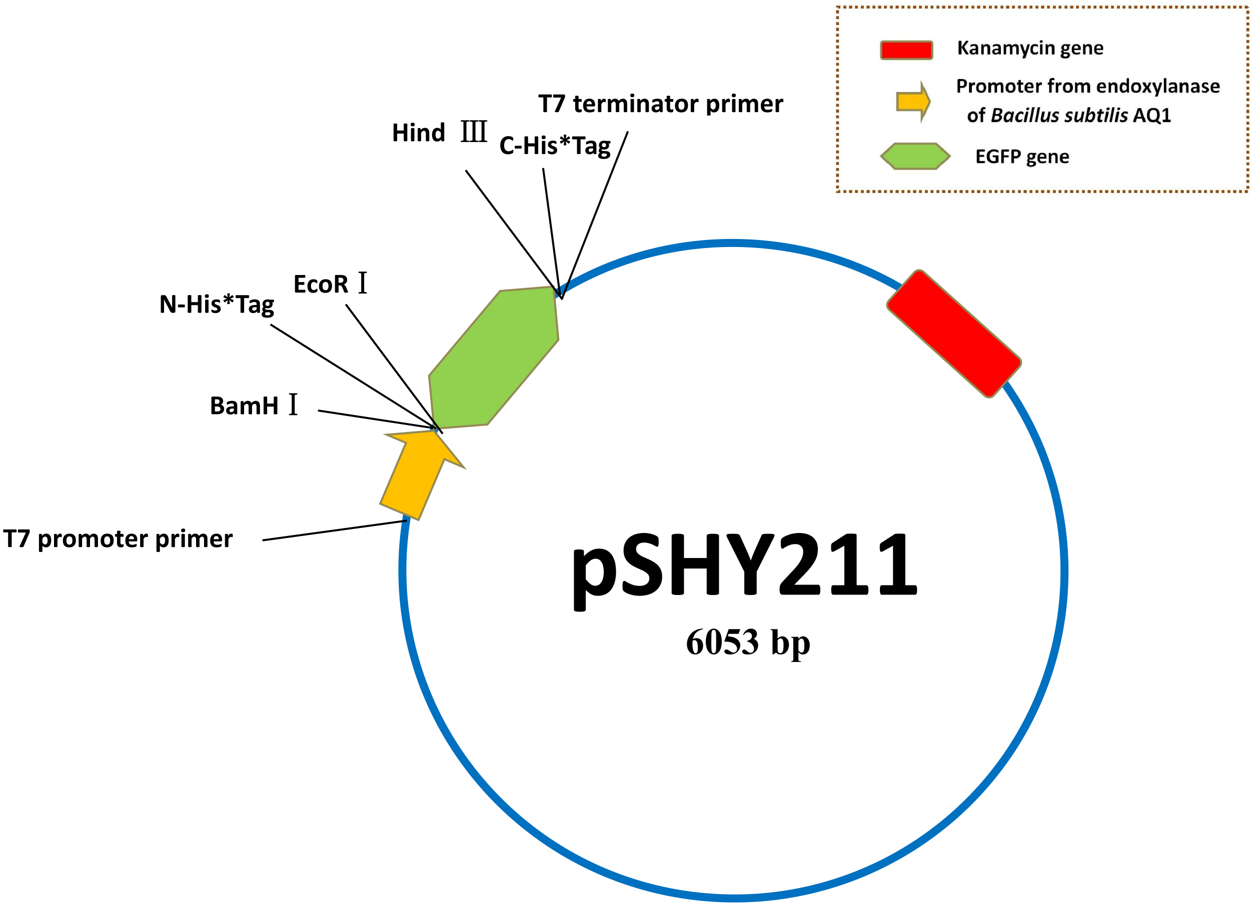

Supplementary Figure S2 The profile of constitutive expression plasmid pSHY211. pSHY211: 6053 bp (check the carrier sequence), the general primers: T7+(5'-TAATACGACTCACTATAGGG-3') and T7ter (5'-GCTAGTTATTGCTCAGCGG-3'). Label: N-His and C-His. Resistance: Kanamycin. Selectable marker gene: UV-enhanced green fluorescent protein gene (*egfp*).

>pSHY211 (The blue DNA sequence is promoter of GH11 endoxylanase gene from *Bacillus subtilis* AQ1. The underlined DNA sequnce are EcoR Ⅰ, BamH Ⅰ and Hind Ⅲ restriction enzyme recognizes sites, respectively. The green DNA sequence is EGFP gene.)

TAATACGACTCACTATAGGG**GGGGTACCTAGCGTGGTATTATACTGAAGGGGACGATCA**
AAAGCGTTGGCGTTCGTTAAATATTTACGAGTGCTGCCTCATGTCAAAGTCAGAAAAA
ATAGTATAGGAGGTAACATATG**GGATCC**GAACATCATCATCATCATCAT**GAATTC**ATGGTG
AGCAAGGGCGAGGAGCTGTTCACCGGGGTGGTGCCCATCCTGGTCGAGCTGGACGGCGAC
GTAAACGGCCACAAGTTCAGCGTGTCCGGCGAGGGCGAGGGCGATGCCACCTACGGCAAG
CTGACCCTGAAGTTCATCTGCACCACCGGCAAGCTGCCCGTGCCCTGGCCCACCCTCGTGA
CCACCCTGACCTACGGCGTGCAGTGCTTCAGCCGCTACCCCGACCACATGAAGCAGCACGA
CTTCTTCAAGTCCGCCATGCCCGAAGGCTACGTCCAGGAGCGCACCATCTTCTTCAAGGAC
GACGGCAACTACAAGACCCGCGCCGAGGTGAAGTTCGAGGGCGACACCCTGGTGAACCG
CATCGAGCTGAAGGGCATCGACTTCAAGGAGGACGGCAACATCCTGGGGCACAAGCTGGA
GTACAACTACAACAGCCACAACGTCTATATCATGGCCGACAAGCAGAAGAACGGCATCAAG

GTGAACTTCAAGATCCGCCACAACATCGAGGACGGCAGCGTGCAGCTCGCCGACCACTAC
CAGCAGAACACCCCCATCGGCGACGGCCCCGTGCTGCTGCCCGACAACCACTACCTGAGC
ACCCAGTCCGCCCTGAGCAAAGACCCCAACGAGAAGCGCGATCACATGGTCCTGCTGGAG
TTCGTGACCGCCGCCGGGATCACTCTCGGCATGGACGAGCTGTACAAGTAA**AAGCTT**GCG
GCCGCACTCGAGCACCACCACCACCACCACTGAGATCCGGCTGCTAACAAAGCCCGAAAG
GAAGCTGAGTTGGCTGCTGCCACCGCTGAGCAATAACTAGCATAACCCCTTGGGGCCTCTA
AACGGGTCTTGAGGGGTTTTTTGCTGAAAGGAGGAACTATATCCGGATTGGCGAATGGGAC
GCGCCCTGTAGCGGCGCATTAAGCGCGGCGGGTGTGGTGGTTACGCGCAGCGTGACCGCTA
CACTTGCCAGCGCCCTAGCGCCCGCTCCTTTCGCTTTCTTCCCTTCCTTTCTCGCCACGTTC
GCCGGCTTTCCCCGTCAAGCTCTAAATCGGGGGCTCCCTTTAGGGTTCCGATTTAGTGCTTT
ACGGCACCTCGACCCCAAAAAACTTGATTAGGGTGATGGTTCACGTAGTGGGCCATCGCCC
TGATAGACGGTTTTTCGCCCTTTGACGTTGGAGTCCACGTTCTTTAATAGTGGACTCTTGTT
CCAAACTGGAACAACACTCAACCCTATCTCGGTCTATTCTTTTGATTTATAAGGGATTTTGCC
GATTTCGGCCTATTGGTTAAAAAATGAGCTGATTTAACAAAAATTTAACGCGAATTTTAACA
AAATATTAACGTTTACAATTTCAGGTGGCACTTTTCGGGGAAATGTGCGCGGAACCCCTATT
TGTTTATTTTTCTAAATACATTCAAATATGTATCCGCTCATGAATTAATTCTTAGAAAAACTCA
TCGAGCATCAAATGAAACTGCAATTTATTCATATCAGGATTATCAATACCATATTTTTGAAAA
AGCCGTTTCTGTAATGAAGGAGAAAACTCACCGAGGCAGTTCCATAGGATGGCAAGATCCT
GGTATCGGTCTGCGATTCCGACTCGTCCAACATCAATACAACCTATTAATTTCCCCTCGTCAA
AAATAAGGTTATCAAGTGAGAAATCACCATGAGTGACGACTGAATCCGGTGAGAATGGCAA
AAGTTTATGCATTTCTTTCCAGACTTGTTCAACAGGCCAGCCATTACGCTCGTCATCAAAAT
CACTCGCATCAACCAAACCGTTATTCATTCGTGATTGCGCCTGAGCGAGACGAAATACGCGA
TCGCTGTTAAAAGGACAATTACAAACAGGAATCGAATGCAACCGGCGCAGGAACACTGCC
AGCGCATCAACAATATTTTCACCTGAATCAGGATATTCTTCTAATACCTGGAATGCTGTTTTC
CCGGGGATCGCAGTGGTGAGTAACCATGCATCATCAGGAGTACGGATAAAATGCTTGATGG
TCGGAAGAGGCATAAATTCCGTCAGCCAGTTTAGTCTGACCATCTCATCTGTAACATCATTG
GCAACGCTACCTTTGCCATGTTTCAGAAACAACTCTGGCGCATCGGGCTTCCCATACAATCG
ATAGATTGTCGCACCTGATTGCCCGACATTATCGCGAGCCCATTTATACCCATATAAATCAGC
ATCCATGTTGGAATTTAATCGCGGCCTAGAGCAAGACGTTTCCCGTTGAATATGGCTCATAA
CACCCCTTGTATTACTGTTTATGTAAGCAGACAGTTTTATTGTTCATGACCAAAATCCCTTAA
CGTGAGTTTTCGTTCCACTGAGCGTCAGACCCCGTAGAAAAGATCAAAGGATCTTCTTGAG
ATCCTTTTTTTCTGCGCGTAATCTGCTGCTTGCAAACAAAAAAACCACCGCTACCAGCGGTG
GTTTGTTTGCCGGATCAAGAGCTACCAACTCTTTTTCCGAAGGTAACTGGCTTCAGCAGAG
CGCAGATACCAAATACTGTCCTTCTAGTGTAGCCGTAGTTAGGCCACCACTTCAAGAACTCT
GTAGCACCGCCTACATACCTCGCTCTGCTAATCCTGTTACCAGTGGCTGCTGCCAGTGGCGA
TAAGTCGTGTCTTACCGGGTTGGACTCAAGACGATAGTTACCGGATAAGGCGCAGCGGTCG
GGCTGAACGGGGGGTTCGTGCACACAGCCCAGCTTGGAGCGAACGACCTACACCGAACTG
AGATACCTACAGCGTGAGCTATGAGAAAGCGCCACGCTTCCCGAAGGGAGAAAGGCGGAC
AGGTATCCGGTAAGCGGCAGGGTCGGAACAGGAGAGCGCACGAGGGAGCTTCCAGGGGG
AAACGCCTGGTATCTTTATAGTCCTGTCGGGTTTCGCCACCTCTGACTTGAGCGTCGATTTT
TGTGATGCTCGTCAGGGGGGCGGAGCCTATGGAAAAACGCCAGCAACGCGGCCTTTTTACG
GTTCCTGGCCTTTTGCTGGCCTTTTGCTCACATGTTCTTTCCTGCGTTATCCCCTGATTCTGT
GGATAACCGTATTACCGCCTTTGAGTGAGCTGATACCGCTCGCCGCAGCCGAACGACCGAG
CGCAGCGAGTCAGTGAGCGAGGAAGCGGAAGAGCGCCTGATGCGGTATTTTCTCCTTACGC

ATCTGTGCGGTATTTCACACCGCATATATGGTGCACTCTCAGTACAATCTGCTCTGATGCCGC
ATAGTTAAGCCAGTATACACTCCGCTATCGCTACGTGACTGGGTCATGGCTGCGCCCCGACA
CCCGCCAACACCCGCTGACGCGCCCTGACGGGCTTGTCTGCTCCCGGCATCCGCTTACAGA
CAAGCTGTGACCGTCTCCGGGAGCTGCATGTGTCAGAGGTTTTCACCGTCATCACCGAAAC
GCGCGAGGCAGCTGCGGTAAAGCTCATCAGCGTGGTCGTGAAGCGATTCACAGATGTCTGC
CTGTTCATCCGCGTCCAGCTCGTTGAGTTTCTCCAGAAGCGTTAATGTCTGGCTTCTGATAA
AGCGGGCCATGTTAAGGGCGGTTTTTTCCTGTTTGGTCACTGATGCCTCCGTGTAAGGGGGA
TTTCTGTTCATGGGGGTAATGATACCGATGAAACGAGAGAGGATGCTCACGATACGGGTTAC
TGATGATGAACATGCCCGGTTACTGGAACGTTGTGAGGGTAAACAACTGGCGGTATGGATG
CGGCGGGACCAGAGAAAAATCACTCAGGGTCAATGCCAGCGCTTCGTTAATACAGATGTAG
GTGTTCCACAGGGTAGCCAGCAGCATCCTGCGATGCAGATCCGGAACATAATGGTGCAGGG
CGCTGACTTCCGCGTTTCCAGACTTTACGAAACACGGAAACCGAAGACCATTCATGTTGTT
GCTCAGGTCGCAGACGTTTTGCAGCAGCAGTCGCTTCACGTTCGCTCGCGTATCGGTGATT
CATTCTGCTAACCAGTAAGGCAACCCCGCCAGCCTAGCCGGGTCCTCAACGACAGGAGCA
CGATCATGCGCACCCGTGGGGCCGCCATGCCGGCGATAATGGCCTGCTTCTCGCCGAAACG
TTTGGTGGCGGGACCAGTGACGAAGGCTTGAGCGAGGGCGTGCAAGATTCCGAATACCGC
AAGCGACAGGCCGATCATCGTCGCGCTCCAGCGAAAGCGGTCCTCGCCGAAAATGACCCA
GAGCGCTGCCGGCACCTGTCCTACGAGTTGCATGATAAAGAAGACAGTCATAAGTGCGGCG
ACGATAGTCATGCCCCGCGCCCACCGGAAGGAGCTGACTGGGTTGAAGGCTCTCAAGGGC
ATCGGTCGAGATCCCGGTGCCTAATGAGTGAGCTAACTTACATTAATTGCGTTGCGCTCACT
GCCCGCTTTCCAGTCGGGAAACCTGTCGTGCCAGCTGCATTAATGAATCGGCCAACGCGCG
GGGAGAGGCGGTTTGCGTATTGGGCGCCAGGGTGGTTTTTCTTTTCACCAGTGAGACGGGC
AACAGCTGATTGCCCTTCACCGCCTGGCCCTGAGAGAGTTGCAGCAAGCGGTCCACGCTG
GTTTGCCCCAGCAGGCGAAAATCCTGTTTGATGGTGGTTAACGGCGGGATATAACATGAGC
TGTCTTCGGTATCGTCGTATCCCACTACCGAGATATCCGCACCAACGCGCAGCCCGGACTCG
GTAATGGCGCGCATTGCGCCCAGCGCCATCTGATCGTTGGCAACCAGCATCGCAGTGGGAA
CGATGCCCTCATTCAGCATTTGCATGGTTTGTTGAAAACCGGACATGGCACTCCAGTCGCCT
TCCCGTTCCGCTATCGGCTGAATTTGATTGCGAGTGAGATATTTATGCCAGCCAGCCAGACG
CAGACGCGCCGAGACAGAACTTAATGGGCCCGCTAACAGCGCGATTTGCTGGTGACCCAAT
GCGACCAGATGCTCCACGCCCAGTCGCGTACCGTCTTCATGGGAGAAAATAATACTGTTGAT
GGGTGTCTGGTCAGAGACATCAAGAAATAACGCCGGAACATTAGTGCAGGCAGCTTCCAC
AGCAATGGCATCCTGGTCATCCAGCGGATAGTTAATGATCAGCCCACTGACGCGTTGCGCG
AGAAGATTGTGCACCGCCGCTTTACAGGCTTCGACGCCGCTTCGTTCTACCATCGACACCA
CCACGCTGGCACCCAGTTGATCGGCGCGAGATTTAATCGCCGCGACAATTTGCGACGGCGC
GTGCAGGGCCAGACTGGAGGTGGCAACGCCAATCAGCAACGACTGTTTGCCCGCCAGTTG
TTGTGCCACGCGGTTGGGAATGTAATTCAGCTCCGCCATCGCCGCTTCCACTTTTTCCCGCG
TTTTCGCAGAAACGTGGCTGGCCTGGTTCACCACGCGGGAAACGGTCTGATAAGAGACAC
CGGCATACTCTGCGACATCGTATAACGTTACTGGTTTCACATTCACCACCCTGAATTGACTCT
CTTCCGGGCGCTATCATGCCATACCGCGAAAGGTTTTGCGCCATTCGATGGTGTCCGGGATC
TCGACGCTCTCCCTTATGCGACTCCTGCATTAGGAAGCAGCCCAGTAGTAGGTTGAGGCCG
TTGAGCACCGCCGCCGCAAGGAATGGTGCATGCAAGGAGATGGCGCCCAACAGTCCCCCG
GCCACGGGGCCTGCCACCATACCCACGCCGAAACAAGCGCTCATGAGCCCGAAGTGGCGA
GCCCGATCTTCCCCATCGGTGATGTCGGCGATATAGGCGCCAGCAACCGCACCTGTGGCGC
CGGTGATGCCGGCCACGATGCGTCCGGCGTAGAGGATCGAGATCTCGATCCCGCGAAAT

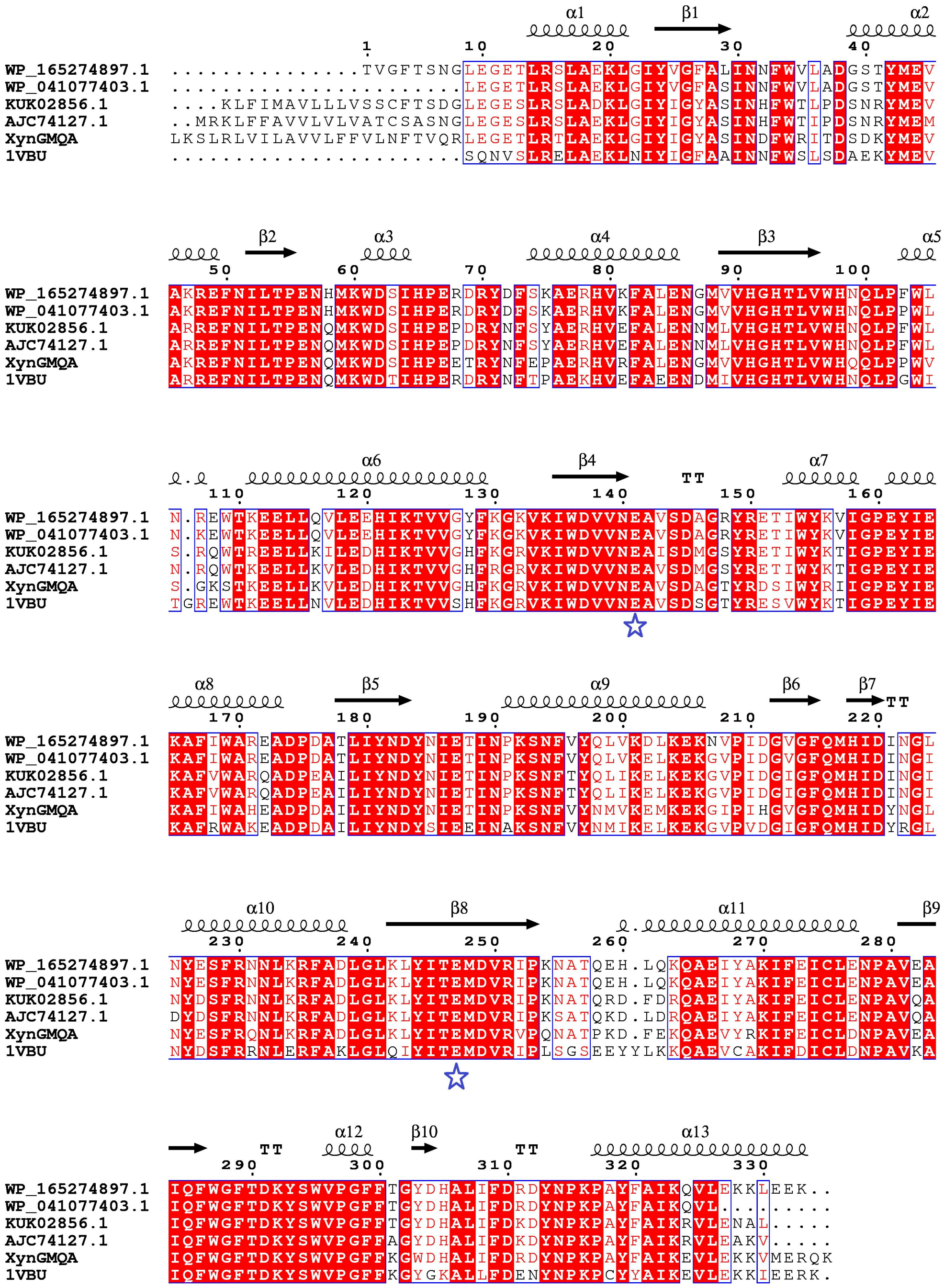

Supplementary Figure S3 Multiple sequence alignment of XynGMQA with some similar xylanases. Two putative catalytic residues (E155 and E262) were marked by blue five-pointed stars.

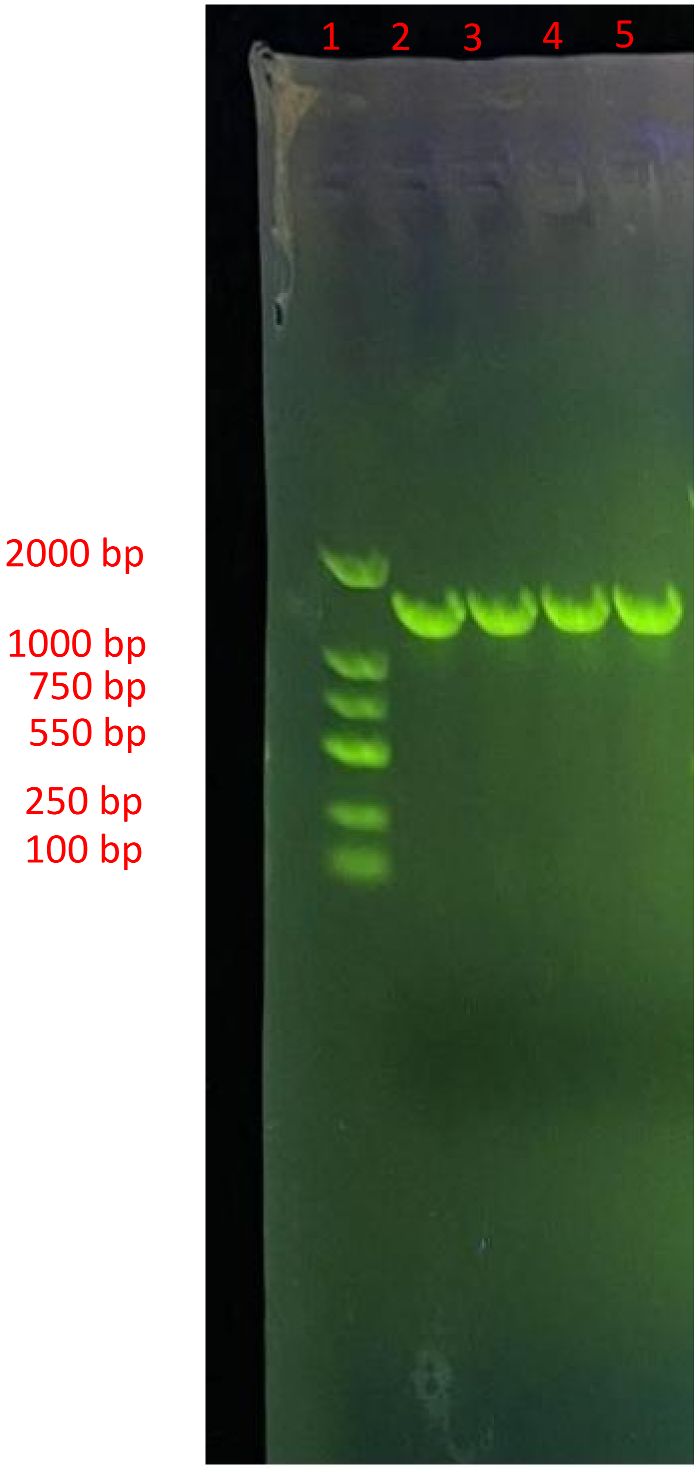

Supplementary Figure S4 Colony PCR results of *E. coli clones* containing xylanase gene (*xyngmqa*). Lane 1, DNA marker, mass indicated on the left; lane 2-5, DNA fragment of *xyngmqA* obtained by PCR amplification.

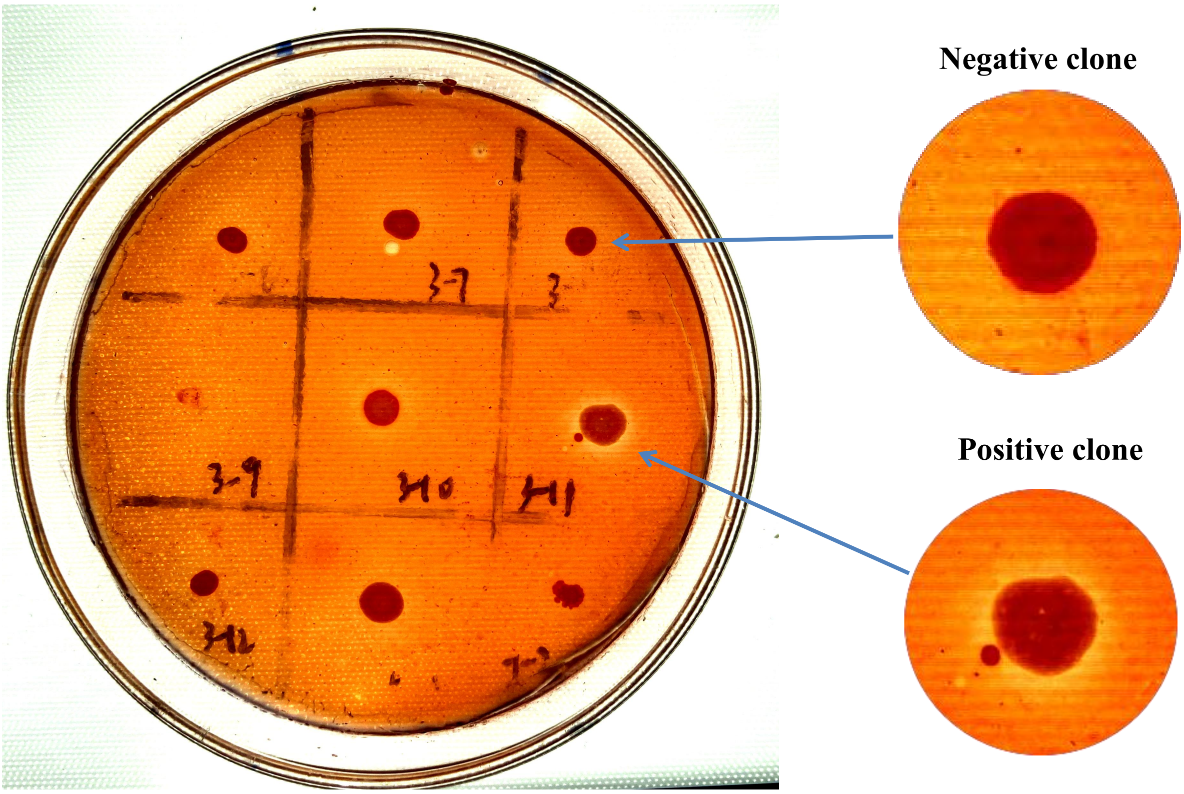

Supplementary Figure S5 Screening of *E. coli* clones containing xylanase gene (*xyngmqa*) by double-layer plate method

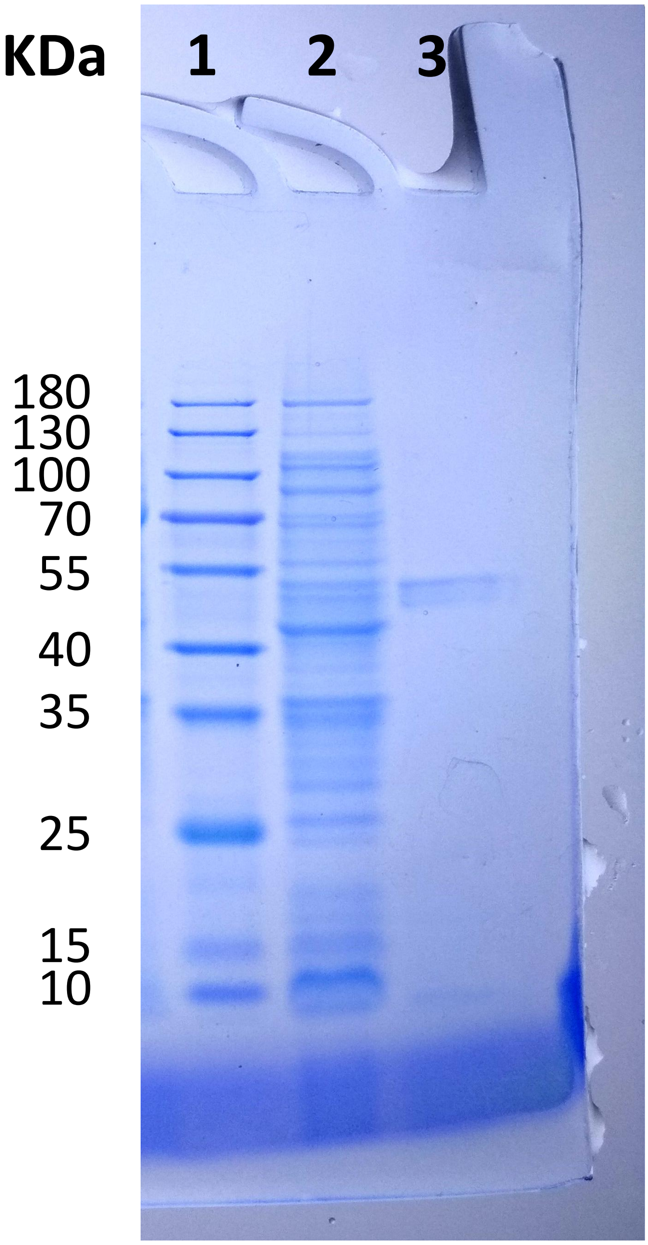

Supplementary Figure S6 SDS-PAGE analysis of XynGMQA of XynGMQA. Lane 1, protein molecular weight marker, mass indicated on the left; lane 2, total protein of *E. coli* DH5α/*pSHY211-XynGMQA*; lane 3, purified XynGMQA.
